# Antidepressant-like Effects of *Garcinia nigrolineata* Resin Extract in a Chronic Mild Stress Mouse Model: Modulation of Monoaminergic and HPA-Axis Pathways

**DOI:** 10.3390/plants14233651

**Published:** 2025-11-30

**Authors:** Yutthana Chotritthirong, Yaowared Sumanont, Supawadee Daodee, Abdulwaris Mading, Chantana Boonyarat, Charinya Khamphukdee, Decha Kumla, Juthamart Maneenet, Kinzo Matsumoto, Anake Kijjoa, Suresh Awale, Orawan Monthakantirat

**Affiliations:** 1 Graduate School of Faculty of Pharmaceutical Sciences, Khon Kaen University, Khon Kaen 40002, Thailand; yutthana_ch@kkumail.com (Y.C.); abdulwaris.m@kkumail.com (A.M.); 2 Division of Pharmaceutical Chemistry, Faculty of Pharmaceutical Sciences, Khon Kaen University, Khon Kaen 40002, Thailand; yaosum@kku.ac.th (Y.S.); csupawad@kku.ac.th (S.D.); chaboo@kku.ac.th (C.B.); 3 Division of Pharmacognosy and Toxicology, Faculty of Pharmaceutical Sciences, Khon Kaen University, Khon Kaen 40002, Thailand; charkh@kku.ac.th; 4 Faculty of Pharmaceutical Sciences, Burapha University, 169 Long Had Bangsaen Road, Chonburi 20131, Thailand; decha.ku@go.buu.ac.th; 5 Natural Drug Discovery Laboratory, Institute of Natural Medicine, University of Toyama, 2630 Sugitani, Toyama 930-0145, Japan; juthamar@inm.u-toyama.ac.jp; 6 Graduate School of Pharmaceutical Sciences, Daiichi University of Pharmacy, Fukuoka 815-8511, Japan; k-matsumoto@daiichi-cps.ac.jp; 7 ICBAS-Instituto de Ciências Biomédicas Abel Salazar and CIIMAR, Universidade do Porto, Rua de Jorge Viterbo Ferreira 228, 4050-313 Porto, Portugal; ankijjoa@icbas.up.pt

**Keywords:** *Garcinia nigrolineata*, chronic mild stress, depression, serotonergic, noradrenergic, HPA-axis

## Abstract

The resin extract of *Garcinia nigrolineata* (GNR-E), a tropical plant used in Southeast Asian traditional medicine, was evaluated for its antidepressant-like effects in a chronic mild stress (CMS) mouse model, with imipramine as a reference drug. GNR-E dose-dependently alleviated CMS-induced anhedonia (sucrose preference test) and behavioral despair (forced swimming and tail suspension tests). Neurochemical analyses revealed that GNR-E increased serotonin (5-HT) and norepinephrine (NE) levels, reduced expression of their transporters (*SERT*, *NET*) and receptors (*5-HT1A*, *1B*, *2A*, *2C*, *7*; *α2A*, 2C) in the frontal cortex and hippocampus, and normalized HPA-axis hyperactivity by lowering serum corticosterone and modulating glucocorticoid receptor (*GR*) and *SGK-1* mRNA expression. In vitro, GNR-E inhibited monoamine oxidase (MAO)-A and -B (*Ki* = 2.33 and 1.55 µg/mL, respectively). Phytochemical analysis identified xanthones, particularly cowanin, as key constituents. These findings highlight GNR-E’s potential as a novel plant-based antidepressant, warranting further investigation into its active compounds and clinical applications.

## 1. Introduction

Major depressive disorder (MDD) is a global public health challenge characterized by disturbances in monoaminergic neurotransmission, which are essential for mood regulation and stress adaptation. Stressful life events can precipitate depression, and individuals with depression often have difficulties in coping with stress. Environmental stressors associated with depression include childhood exposure to adversity, acute life events, and chronic stress [1,2,3]. Chronic exposure to stress has been shown to dysregulate the monoaminergic systems such as serotonin (5-HT) and norepinephrine (NE) systems, including their receptors (e.g., *5-HT1A*, *1B*, *2A*, *2C*, *7*; *α2A*, *α2C*) and trans-porters (SERT, NET), and activate the hypothalamic–pituitary–adrenal (HPA) axis, leading to increased corticosterone (CORT) levels, hippocampal dysfunction, and behavioral despair [4,5,6,7].

Although current antidepressant drugs that target monoaminergic systems (e.g., selective serotonin reuptake inhibitors) can alleviate symptoms, their delayed onset and limited efficacy underscore the need for novel therapeutic agents with multiple mechanisms of action [8,9].

Natural xanthone derivatives have emerged as promising candidates due to their antioxidant, anti-inflammatory, and neuroprotective effects [10,11]. *Garcinia nigrolineata* Planch. ex T. Anderson (Clusiaceae), known in Thailand as “Cha-Muang,” is traditionally used for treating fever, indigestion, and respiratory ailments [12,13,14]. Its resin, rich in xanthones (e.g., cowanin and α-mangostin), exhibits antioxidant, anti-inflammatory, cytotoxicity, and neuroprotective properties [15,16,17].

While α-mangostin has shown antidepressant-like properties through monoaminergic modulation [17], the antidepressant potential of *G. nigrolineata* resin extract (GNR-E) under chronic mild stress (CMS) model remains unexplored. Our prior work demonstrated GNR-E’s effects on stress-induced memory deficits and monoamine oxidase (MAO) inhibition [14], suggesting its therapeutic potential.

This study aims to evaluate the antidepressant-like effects of GNR-E within a CMS mouse model. The investigation focuses on the modulation of serotonergic and noradrenergic biomarkers in the frontal cortex (FC) and hippocampus (HP), alongside the regulation of HPA axis activity, serum CORT levels, and GR signaling. This study seeks to establish GNR-E as an innovative plant-based therapeutic intervention for MDD.

## 2. Results

### 2.1. Monoamine Oxidase A and B (MAO-A and MAO-B) Inhibitory Activity of GNR-E

The CH_2_Cl_2_ extract of *Garcinia nigrolineata* resin (GNR-E) was evaluated for its inhibitory effects on monoamine oxidase A (MAO-A) and B (MAO-B) activities. GNR-E exhibited dose-dependent inhibition, with enzyme-inhibitor dissociation constant (*Ki*) values of 2.33 µg/mL for MAO-A and 1.55 µg/mL for MAO-B, calculated using the Cheng-Prusoff equation (*Ki* = IC_50_/(1 + [S]/*Km*)) (Table 1). GNR-E exhibited potent inhibition of both MAO isoforms, with IC_50_ values of 8.81 ± 0.02 µg/mL for MAO-A and 3.60 ± 0.04 µg/mL for MAO-B, indicating approximately 2.4-fold greater potency against MAO-B compared to MAO-A. This preferential inhibition of MAO-B, a key molecular target of the anti-Parkinson drug deprenyl, suggests potential therapeutic relevance for neurodegenerative disorders. The *Ki* values of GNR-E further supported its stronger binding affinity for MAO-B compared to MAO-A, with a selectivity index (Si) of 0.67 for MAO-B and 1.50 for MAO-A, reinforcing its MAO-B preference. The effect of GNR-E’s antidepressant-like effects and its efficacy was further assessed in the chronic unpredictable mild stress (CMS) model of depression. 

### 2.2. The Effect of GNR-E on Anhedonia Behavior in the Sucrose Preference Test

**Figure 1:**
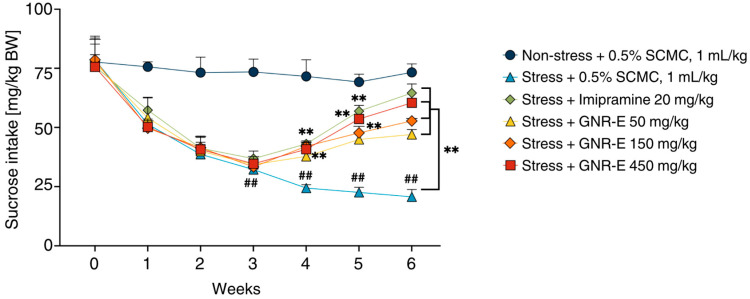
The effect of the GNR-E on anhedonia behavior in the sucrose preference test using CMS mice. The amount of 2% sucrose solution taken by each animal group was measured as an index of sucrose preference test once a week. Each data point represents the mean ± SEM (n = 12). ^##^ *p* < 0.001 vs. the vehicle-treated non-stress group. ** *p* < 0.001 vs. the vehicle-treated CMS group (post hoc Tukey test).

The sucrose preference test was conducted to assess anhedonia-like behavior induced by chronic unpredictable mild stress (CMS) in mice. Sucrose consumption, measured weekly as an index of preference for a 2% sucrose solution, was significantly reduced in the vehicle-treated CMS group compared to the non-stress group (*p* < 0.001, post hoc Tukey test; Figure 1). In contrast, CMS mice treated with imipramine exhibited a significant increase in sucrose intake by week 4. Similarly, CMS mice administered GNR-E at doses of 50, 150, and 450 mg/kg showed a marked increase in sucrose consumption by week 6 compared to the vehicle-treated CMS group (*p* < 0.001, post hoc Tukey test). Data are presented as mean ± SEM (n = 12), with detailed statistical analyses available in Supplementary Table S1. These findings suggest that GNR-E effectively reverses CMS-induced anhedonia in a dose-dependent manner, with effects comparable to the reference antidepressant imipramine.

### 2.3. Effect of GNR-E on CMS-Induced Learned Helplessness Behavior in Forced Swimming Test and Tail Suspension Test

**Figure 2:**
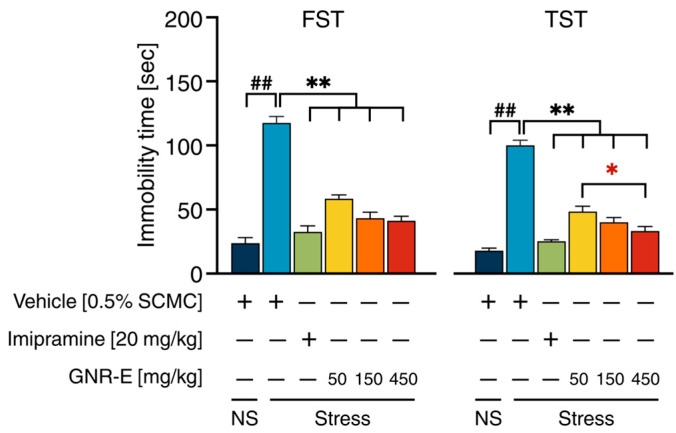
The ameliorative effects of GNR-E on CMS-induced behavioral despair in FST (**left**) and TST (**right**). Each column illustrates the mean ± SEM (n = 12). ^##^ *p* < 0.001 vs. the vehicle-treated non-stress group (NS). ** *p* < 0.001 vs. the vehicle-treated CMS group. * *p* < 0.05, red asterisks indicate comparisons in different doses of GNR-E (post hoc Tukey test).

The Forced Swimming Test (FST) and Tail Suspension Test (TST) were employed to evaluate CMS-induced depressive-like behaviors in mice. In both tests, vehicle-treated CMS mice exhibited a significant increase in immobility time compared to the non-stress control group (*p* < 0.001, post hoc Tukey test; Figure 2). Treatment with GNR-E at doses of 50, 150, and 450 mg/kg significantly reduced immobility time in both FST and TST, with effects comparable to those observed in imipramine-treated CMS mice (*p* < 0.001 vs. vehicle-treated CMS group). In FST (Figure 2, left), GNR-E at 450 mg/kg showed the most pronounced reduction, while in TST (Figure 2, right), a dose-dependent decrease was evident, with a significant difference between 50 and 450 mg/kg doses (*p* < 0.05). Data are presented as mean ± SEM (n = 12), with detailed statistical analyses provided in Supplementary Tables S2 and S3. These results indicate that GNR-E effectively ameliorates CMS-induced behavioral despair in a dose-dependent manner.

### 2.4. Effect of GNR-E on CMS-Induced Increase in Serum Corticosterone Levels

**Figure 3:**
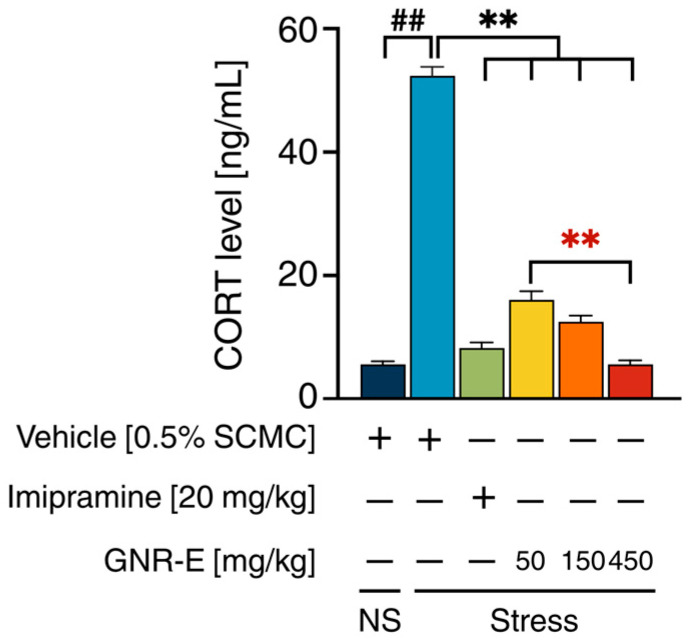
The effects of GNR-E and imipramine (IMP) on CMS-induced elevation of serum CORT levels. Each column represents the mean ± SEM (n = 6). ^##^ *p* < 0.001 vs. the vehicle-treated non-stress group (NS). ** *p* < 0.001 vs. the vehicle-treated CMS group. ** *p* < 0.001, red asterisks indicate comparisons in different doses of GNR-E (post hoc Tukey test).

The impact of GNR-E and imipramine (IMP) on serum corticosterone (CORT) levels was assessed in CMS-exposed mice. Vehicle-treated CMS mice exhibited significantly elevated serum CORT levels compared to the non-stress control group (*p* < 0.001, post hoc Tukey test; Figure 3). Daily administration of imipramine (20 mg/kg) and GNR-E at doses of 50, 150, and 450 mg/kg significantly reduced these elevated CORT levels (*p* < 0.001 vs. vehicle-treated CMS group). Notably, a dose-dependent decrease was observed with GNR-E, with the 450 mg/kg dose showing the most substantial reduction (*p* < 0.001 compared between doses). Data are presented as mean ± SEM (n = 6), with detailed statistical analyses provided in Supplementary Table S4. These findings suggest that GNR-E effectively mitigates CMS-induced hypercortisolemia in a dose-dependent manner, similar to imipramine.

### 2.5. Effect of GNR-E on CMS-Induced Decrease in 5-HT and NE Levels

**Figure 4:**
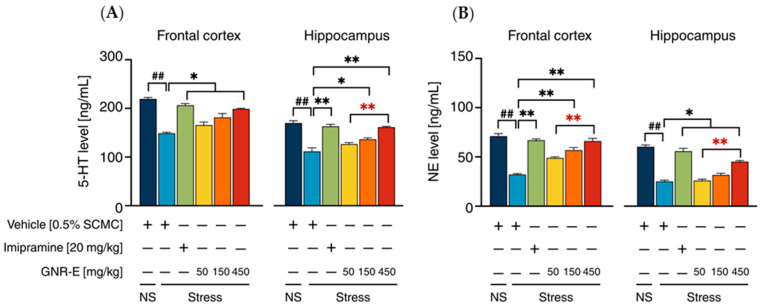
The effects of GNR-E and imipramine (IMP) on CMS-induced decrease in 5-HT (**A**) and NE (**B**) levels in the frontal cortex and hippocampus. Each data column represents the mean ± SEM (n = 6). ^##^ *p* < 0.001 vs. the vehicle-treated non-stress group (NS). * *p* < 0.05 and ** *p* < 0.001 vs. the vehicle-treated CMS group. ** *p* < 0.001, red asterisks indicate comparisons in different doses of GNR-E (post hoc Tukey test).

The effect of GNR-E and imipramine (IMP) on serotonin (5-HT) and norepinephrine (NE) levels was evaluated in the frontal cortex (FC) and hippocampus (HP) of CMS-exposed mice. Vehicle-treated CMS mice displayed significant reductions in 5-HT (Figure 4A) and NE (Figure 4B) levels compared to the non-stress group (*p* < 0.001, post hoc Tukey test). Daily administration of imipramine (20 mg/kg) and GNR-E at 450 mg/kg significantly restored 5-HT and NE levels in both brain regions (*p* < 0.05, *p* < 0.001 vs. vehicle-treated CMS group). GNR-E at lower doses (50 and 150 mg/kg) showed a dose-dependent increase, with 150 mg/kg reaching significance in the HP for 5-HT (*p* < 0.001 between doses) and FC for NE (*p* < 0.05 between doses). Non-significant differences (ns) were observed between some groups, particularly at lower GNR-E doses. Data are presented as mean ± SEM (n = 6) with detailed statistical analyses in Supplementary Tables S5 and S6. These results indicate that GNR-E effectively reverses CMS-induced neurotransmitter depletion in a dose-dependent manner, with efficacy comparable to imipramine at the highest dose.

### 2.6. Effect of GNR-E on CMS-Induced Changes in Gene Expression

**Figure 5:**
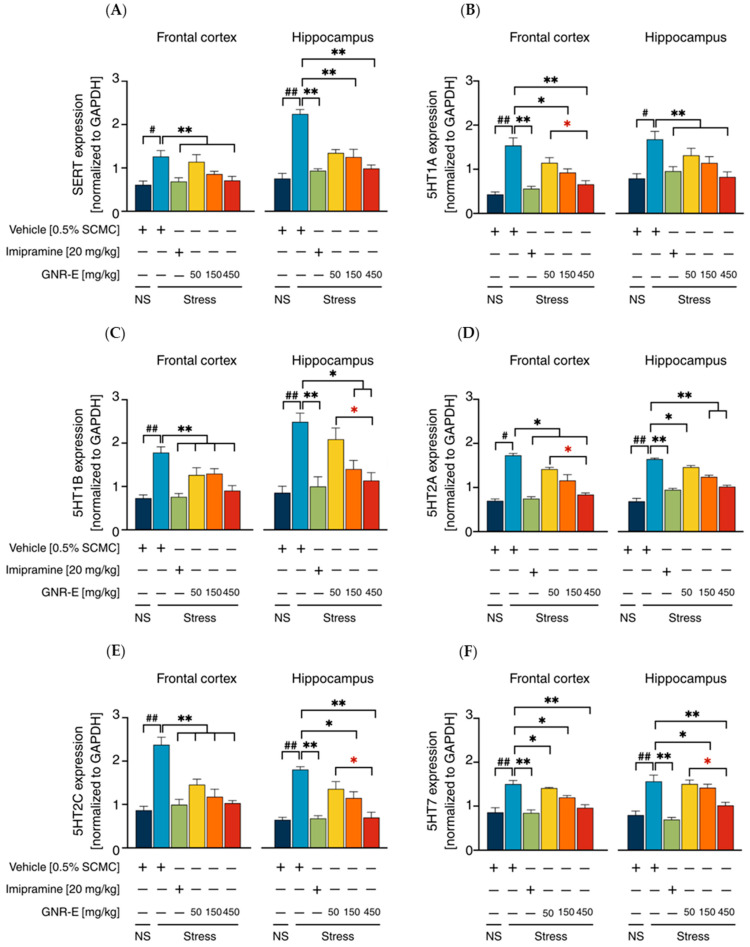
Effects of GNR-E and imipramine (IMP) on CMS-induced changes in expression levels of genes encoding *SERT* (**A**), *5HT1A* (**B**), *5HT1B* (**C**), *5HT2A* (**D**), *5HT2C* (**E**), and *5HT7* (**F**) in the frontal cortex and hippocampus. Each column represents the mean ± SEM (n = 6). ^#^ *p* < 0.05, ^##^ *p* < 0.001 vs. the vehicle-treated non-stress group (NS). * *p* < 0.05, ** *p* < 0.001 vs. the vehicle-treated CMS group. * *p* < 0.05, red asterisks indicate comparisons in different doses of GNR-E (post hoc Tukey test).

**Figure 6:**
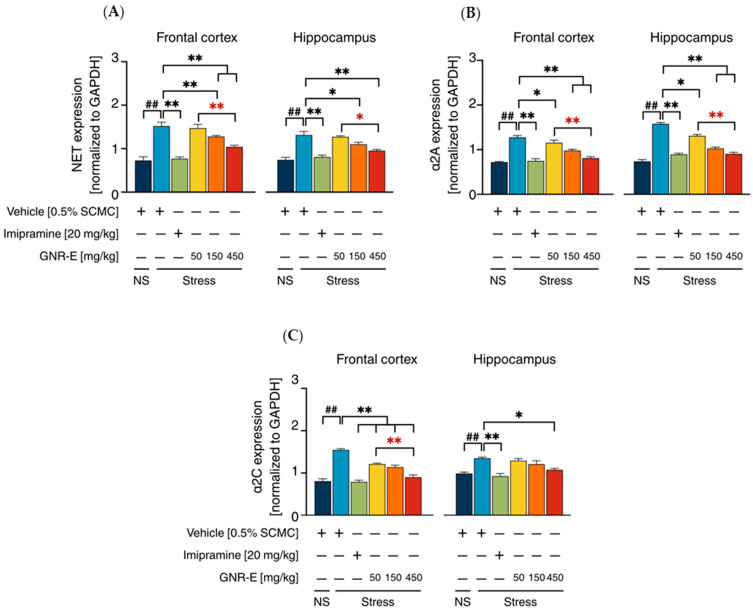
Effects of daily administration of GNR-E and imipramine (IMP) on CMS-induced changes expression levels of genes encoding *NET* (**A**), *α2A* (**B**), and *α2C* (**C**) in the frontal cortex and hippocampus. Each column represents the mean ± SEM (n = 6). ^##^ *p* < 0.001 vs. the vehicle-treated non-stress group (NS). * *p* < 0.05, ** *p* < 0.001 vs. the vehicle-treated CMS group. * *p* < 0.05, ** *p* < 0.001 red asterisks indicate comparisons in different doses of GNR-E (post hoc Tukey test).

Serotonergic and Noradrenergic Systems: The expression of genes encoding serotonergic biomarkers (*SERT*, *5-HT1A*, *5-HT1B*, *5-HT2A*, *5-HT2C*, and *5-HT7*) and noradrenergic biomarkers (*NET*, *α2A*, and *α2C*) was analyzed using quantitative real-time PCR (qPCR) in the frontal cortex (FC) and hippocampus (HP) of CMS-exposed mice. Vehicle-treated CMS mice exhibited significant upregulation of these genes compared to the non-stress group (*p* < 0.001, *p* < 0.05, post hoc Tukey test; Figure 5A–F). Daily administration of imipramine (20 mg/kg) and GNR-E at 50, 150, and 450 mg/kg significantly reduced these elevations (*p* < 0.05, *p* < 0.001 vs. vehicle-treated CMS group), with a dose-dependent effect most pronounced at 450 mg/kg (*p* < 0.05 between doses). Some lower-dose comparisons showed non-significant differences (ns). Similarly, for noradrenergic genes (*NET*, *α2A*, *α2C*; Figure 6), CMS increased expression (*p* < 0.001), which was significantly attenuated by imipramine and GNR-E, with the highest dose showing the greatest effect (*p* < 0.05, *p* < 0.001 between doses).

**Figure 7:**
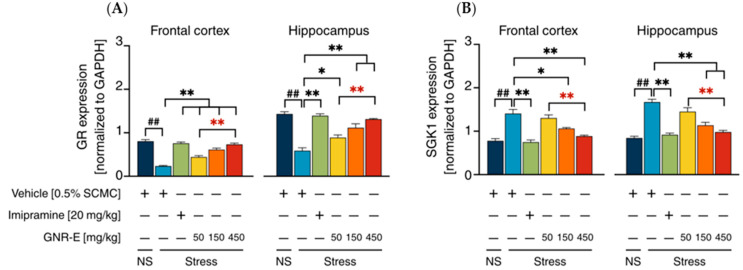
Effects of daily GNR-E and imipramine (IMP) administrations on CMS-induced changes in expression levels of genes encoding glucocorticoid receptor (*GR*) (**A**) and glucocorticoid-inducible kinase-1 (*SGK-1*) (**B**) in the frontal cortex and hippocampus. Each column represents the mean ± SEM (n = 6). ^##^ *p* < 0.001 vs. the vehicle-treated non-stress group (NS). * *p* < 0.05, ** *p* < 0.001 vs. the vehicle-treated CMS group. ** *p* < 0.001, red asterisks indicate comparisons in different doses of GNR-E (post hoc Tukey test).

HPA-Axis System: The expression of genes encoding glucocorticoid receptor (*GR*) and serum/glucocorticoid-regulated kinase-1 (*SGK-1*), markers of HPA-axis activity, was assessed in FC and HP. CMS exposure significantly increased *SGK-1* mRNA levels (*p* < 0.001) and decreased *GR* mRNA levels (*p* < 0.001) compared to the non-stress group (Figure 7A,B). Daily treatment with imipramine and GNR-E at 50, 150, and 450 mg/kg significantly reversed these changes (*p* < 0.05, *p* < 0.001 vs. vehicle-treated CMS group), with GNR-E showing a dose-dependent effect, particularly at 450 mg/kg (*p* < 0.001 between doses, *p* < 0.05 between doses). Data are presented as mean ± SEM (n = 6), with detailed statistical analyses in Supplementary Tables S7–S17. These results indicate that GNR-E modulates CMS-induced alterations in serotonergic, noradrenergic, and HPA-axis gene expression in a dose-dependent manner, comparable to imipramine.

### 2.7. Phytochemical Profiling and HPLC-Based Identification of Major Constituents in GNR-E

The total xanthone, phenolic, and flavonoid contents of GNR-E were determined using calibration curves with correlation coefficients of r^2^ = 0.9981, r^2^ = 0.9996, r^2^ = 0.9999, respectively. These contents were quantified as equivalents of α-mangostin (α-MG), gallic acid (GAE), and quercetin (QE), respectively, and are summarized in Table 2. The GNR-E extract contained 156.24 ± 0.22 mg α-MG/g, 103.12 ± 0.72 mg GAE/g, and 80.79 ± 0.51 mg QE/g, indicating a high concentration of xanthones, followed by phenolic compounds and flavonoids.

The chemical composition of *Garcinia nigrolineata* resin extract (GNR-E) was characterized using reversed-phase high-performance liquid chromatography (HPLC) coupled with diode-array detection (DAD). Five marker compounds—cowagarcinone C (**1**), cowaxanthone (**2**), α-mangostin (**3**), 7-*O*-methylgarcinone E (**4**), and cowanin (**5**)—were employed as reference standards, all previously isolated from GNR-E [14].Calibration curves were constructed for each compound using appropriate concentration ranges: 1–20 µg/mL for **1** and **3**; 5–30 µg/mL for **2** and **5**; and 0.5–15 µg/mL for **4**. Standard solutions were prepared from a 1 mg/mL stock solution. For sample preparation, 10 mg of GNR-E was dissolved in methanol to obtain a 10 mg/mL stock solution, which was subsequently diluted with the mobile phase to the desired concentrations. All solutions were filtered through a 0.45 µm nylon syringe filter prior to HPLC injection.

**Figure 8:**
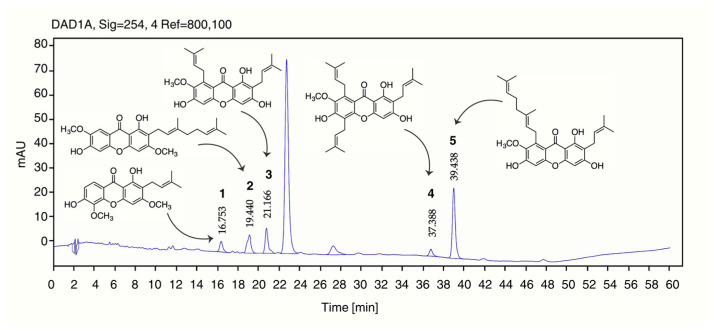
Chromatographic profile of GNR-E. Peaks corresponds to cowagarcinone C (**1**), cowaxanthone (**2**), α-mangostin (**3**), 7-*O*-methylgarcinone E (**4**), and cowanin (**5**) of GNR-E.

The analytical method was validated in accordance with the International Council for Harmonisation (ICH) Q2(R2) guidelines [18]. The method exhibited excellent linearity (R^2^ > 0.99) for all analytes, with high precision demonstrated by intra-day and inter-day relative standard deviations (%RSDs) below 3%. Accuracy, assessed through recovery experiments, ranged from 95% to 108%, indicating strong agreement between measured and true concentrations. Quantitative analysis confirmed the presence of all five target compounds in GNR-E, with retention times consistent with those of the reference standards. The concentration of each compound, expressed in mg/g of extract, is summarized in Table 2, and the corresponding chromatographic profiles are presented in Figure 8.

## 3. Discussion

Chronic mild stress (CMS) serves as a validated animal model for depression, exhibiting construct validity through shared mechanisms, face validity via behavioral symptoms resembling human depression, and predictive validity through responsiveness to antidepressants. This study demonstrates that daily GNR-E administration mitigates behavioral and neurochemical alterations associated with depression in this model. In this study, male mice were used exclusively to reduce variability associated with the estrous cycle, which can affect behavioral and neurochemical outcomes in depression models. While this approach improves experimental consistency, we acknowledge that future studies should include female mice to evaluate potential sex-specific effects of GNR-E.

Initial investigations focused on GNR-E’s effects on monoamine oxidase (MAO) isoforms, MAO-A and MAO-B, which degrade 5-HT, NE, and DA—key neurotransmitters in mood and cognition regulation. MAO-A preferentially metabolizes 5-HT and NE, inhibited by clorgyline (an antidepressant), while MAO-B targets DA, inhibited by deprenyl (an anti-Parkinson agent enhancing L-DOPA effects). Results show GNR-E inhibits MAO-B approximately 2.4-fold more selectively than MAO-A in vitro, aligning with prior findings on *Garcinia* spp. extracts rich in xanthones, such as *G. indica*, which reduce brain MAO levels. Previous studies identified GNR-E constituents (e.g., cowaxanthone, cowanin, xanthochymusxanthone A, cowagarcinone A) selectively inhibiting MAO-A, and α-mangostin and cowaxanthone B targeting MAO-B. This dual inhibitory profile suggests GNR-E’s potential to modulate mood and cognition, prompting further exploration of its antidepressant effects.

In the CMS model, vehicle treated male mice were used exclusively displayed reduced sucrose consumption, indicative of anhedonia—a hallmark of major depressive disorder (MDD). Daily GNR-E and imipramine administration from week 4 significantly increased sucrose intake, with GNR-E’s effect emerging dose- and duration-dependently by week 6, mirroring the delayed onset (≥2 weeks) of conventional antidepressants. Additionally, GNR-E reduced immobility time in the forced swim test (FST) and tail suspension test (TST), effects comparable to imipramine and xanthone derivatives from the Clusiaceae family. Sahu et al. reported α-mangostin from *G. mangostana* reducing immobility by elevating 5-HT, supporting the role of xanthones in GNR-E’s antidepressant-like behavioral effects.

Mechanistically, CMS reduced 5-HT and NE levels in the frontal cortex (FC) and hippocampus (HP), which GNR-E and imipramine restored, suggesting enhanced serotonergic and noradrenergic neurotransmission. CMS upregulated *SERT* and *NET* mRNA expression in these regions, consistent with reduced synaptic 5-HT and NE availability, a trend reversed by imipramine and GNR-E (150–450 mg/kg). This normalization likely contributes to GNR-E’s antidepressant effects, paralleling imipramine’s mechanism.

Regarding serotonin receptors, CMS increased *5-HT1A* and *5-HT1B* mRNA expression in FC and HP, which GNR-E (450 mg/kg) and imipramine reversed, aligning with reports of *5-HT1A* downregulation by desipramine and *5-HT1B*’s role in autoregulation. CMS also elevated *5-HT2A*, *5-HT2C*, and *5-HT7* receptor expression, mitigated by GNR-E (150–450 mg/kg) and imipramine, consistent with studies linking these receptors to depression-like behaviors and their reversal by antagonists. For noradrenergic systems, CMS upregulated *α2A* and *α2C* receptor expression, reversed by GNR-E (150–450 mg/kg) and imipramine, supporting their role as therapeutic targets, as evidenced by prior stress and post-mortem studies. GNR-E is a phytochemical mixture enriched in xanthone derivatives, predominantly compound (cowanin), with minor components such as α-mangostin, cowaxanthone, cowagarcinone C, and 7-*O*-methylgarcinone E. Our previous study revealed that α-mangostin showed stronger MAO-B inhibition, while cowanin was the most potent toward MAO-A. Additionally, SAR evidence indicates that hydroxyl groups at C-1 and C-3 and prenyl groups at C-2 and C-8 play crucial roles in α-mangostin’s MAO-A and MAO-B inhibitory activity [19,20].

HPA-axis dysregulation was evident with CMS-induced serum corticosterone (CORT) elevation and increased *SGK-1* mRNA, alongside reduced *GR* mRNA expression in FC and HP. GNR-E dose-dependently lowered CORT levels, and both GNR-E and imipramine reversed *SGK-1* and *GR* expression changes, suggesting HPA-axis modulation. This aligns with evidence that excessive *GR* activation via high CORT upregulates *SGK-1*, impairing neurogenesis, a process GNR-E may counteract.

The phytochemical analysis of *Garcinia nigrolineata* resin extract (GNR-E) revealed five key compounds: cowagarcinone C (**1**), cowaxanthone (**2**), α-mangostin (**3**), 7-*O*-methylgarcinone E (**4**), and cowanin (**5**). Among these, 5 is likely the principal bioactive xanthone derivative responsible for the observed MAO inhibition and neurotransmitter modulation, although contributions from the other identified compounds cannot be excluded. The peak observed at Rt 23 min in the chromatographic profile of GNR-E could not be matched to any of our previously purified isolates, indicating that this component was not detected under the present separation conditions. Future study will include LC-MS analysis to elucidate this peak and expand the complete chemical profile of GNR-E, guiding further bioactivity investigations.

**Figure 9:**
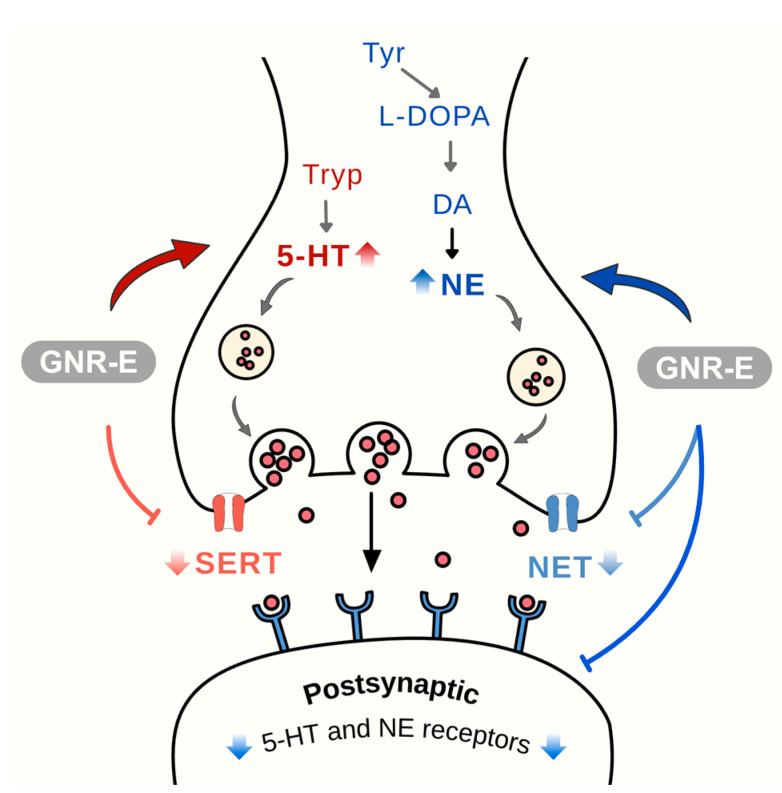
Effects of *Garcinia nigrolineata* resin extract (GNR-E) on serotonergic and noradrenergic modulation in depression. Schematic representation of serotonin (5-HT) and norepinephrine (NE) synapses showing possible effect on neurotransmission pathways, receptor subtypes (5-HT1A, 5-HT1B, 5-HT2A, 5-HT2C, 5-HT7 for serotonin; α2A and α2C for norepinephrine), and reuptake transporters (SERT and NET). Abbreviations: 5-HT, serotonin; NE, norepinephrine; SERT, serotonin transporter; NET, norepinephrine transporter. Sample size: n = 6 per group for neurochemical and gene expression analysis. Scale bar: not applicable (schematic illustration).

**Figure 10:**
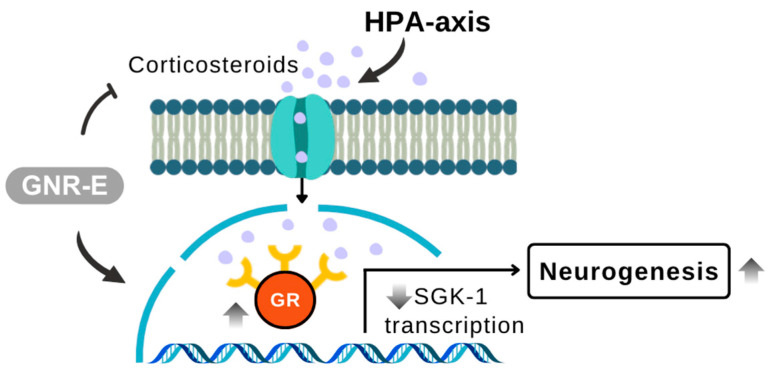
Schematic illustration showing the modulation of the hypothalamic–pituitary–adrenal (HPA) axis components in response to GNR-E treatment. GNR-E attenuates corticosterone-induced overactivation of glucocorticoid receptor (GR) signaling and downregulates SGK-1 transcription, contributing to restored neurogenesis. Abbreviations: GR, glucocorticoid receptor; SGK-1, serum/glucocorticoid-regulated kinase 1; HPA-axis, hypothalamic–pituitary–adrenal axis. Sample size: n = 6 per group for serum corticosterone and mRNA expression analysis. Scale bar: not applicable (schematic illustration).

In conclusion, GNR-E exhibits antidepressant-like effects in the CMS model by targeting monoaminergic systems (Figure 9) and the HPA axis (Figure 10), key pathways affected by chronic stress. Its efficacy, comparable to imipramine, and rich phytochemical profile highlight its potential as a therapeutic option from Thai traditional medicine for mood disorders.

Despite the novel findings presented in this study, several limitations should be acknowledged. The effective dose of GNR-E (450 mg/kg) is relatively high, and comprehensive toxicity assessments (acute and chronic) were not conducted. The mechanistic insights are primarily descriptive, relying on mRNA expression and neurotransmitter quantification, without validation at the protein level or causal verification using receptor-specific pharmacological tools. Future studies should aim to address these limitations by validating protein expression of key markers in monoaminergic and HPA-axis pathways, and by incorporating selective receptor blockers (e.g., *5-HT2A* or *5-HT3* antagonists) to clarify the specific neurochemical mechanisms through which GNR-E exerts its antidepressant effects. Additionally, further research should include comprehensive toxicological assessments to establish the safety profile of GNR-E, followed by clinical investigations to evaluate the therapeutic potential of the extract and its xanthone constituents in humans.

## 4. Materials and Methods

### 4.1. Plant Material, Extraction, Phytochemical Profiling, and HPLC Fingerprint

#### 4.1.1. Plant Material and Extraction

*Garcinia nigrolineata* Planch. ex T. Anderson (Clusiaceae) was collected from the Botanical gardens, Faculty of Pharmaceutical Sciences, Khon Kaen University, Khon Kaen, Thailand, in June 2022. Assistant Professor Dr. Prathan Luecha taxonomically identified the plant material, and a voucher specimen (PSKKU-PL-047) was deposited at the Herbarium of the Faculty of Pharmaceutical Sciences, Khon Kaen University. Dried resin (2.96 kg) of *G. nigrolineata* was subjected to microwave-assisted extraction, and isolation of phytochemical constituents was performed as previously described [14].

#### 4.1.2. Phytochemical Profiling

The total xanthone content (TXC) of *Garcinia nigrolineata* resin extract (GNR-E) was quantified using a modified UV–Vis spectrophotometric method [20]. GNR-E (20 µg/mL in 80% methanol) was measured at 245 nm, and TXC was calculated from a standard curve of α-mangostin (0–200 µg/mL), expressed as mg α-MG/g extract.

Total phenolic content (TPC) was determined via the Folin–Ciocalteu assay, using gallic acid as a standard and expressed as mg GAE/g extract [21]. Total flavonoid content (TFC) was assessed by the aluminum chloride method, with quercetin as a reference, and reported as mg QE/g extract.

#### 4.1.3. HPLC Fingerprint of the GNR-E

The chemical profiling of the GNR-E was conducted using reversed-phase high-performance liquid chromatography (HPLC) equipped with a Hypersil ODS column (Agilent Technologies Inc., Santa Clara, CA, USA; 4 × 250 mm, 5 µm). A gradient elution was performed using methanol (35–65%) and 0.1% formic acid in ultrapure water at a flow rate of 1 mL/min. The analysis was carried out over a 60 min runtime, and UV detection was monitored at 245 nm. Standard reference compounds isolated from GNR-E included cowagarcinone C (**1**), cowaxanthone (**2**), α-mangostin (**3**), 7-*O*-methylgarcinone E (**4**), and cowanin (**5**).

Method validation was performed following the International Council for Harmonisation (ICH) Q2(R2) guidelines [18]. Calibration curves were constructed for quantification. Compounds **1** and **3**, standard concentrations of 1, 2.5, 5, 10, 15, and 20 µg/mL were used. For compounds **2** and **5**, the curves were prepared using concentrations of 5, 10, 15, 20, 25, and 30 µg/mL. Compound **4**, standard concentrations were 0.5, 1, 2.5, 5, 10, and 15 µg/mL. All standard solutions were prepared from a 1 mg/mL stock. For sample preparation, 10 mg of GNR-E was dissolved in methanol to obtain a stock solution (10 mg/mL), which was then diluted using the mobile phase to the appropriate concentration. Prior to injection, all solutions were filtered through a 0.45 µm nylon syringe filter.

### 4.2. MAO-A and MAO-B Inhibitory Assay

Recombinant human MAO-A and MAO-B (Sigma-Aldrich, St. Louis, MO, USA) were assayed using clorgyline and deprenyl (Sigma-Aldrich, St. Louis, MO, USA) as standard inhibitors for MAO-A and MAO-B, respectively. GNR-E (100 mg) was dissolved in DMSO (400 µL) to prepare a 250 mg/mL stock solution, followed by serial dilutions. Each sample concentration (20 µL) was mixed with kynuramine (9 µL for MAO-A, 6 µL for MAO-B; Sigma-Aldrich, St. Louis, MO, USA) and potassium phosphate buffer (PBS, 100 mM, pH 7.4; 469.5 µL for MAO-A, 472.5 µL for MAO-B). MAO-A or MAO-B enzyme (1.5 µL) was added, and the mixture was incubated at 37 °C for 20 min. The reaction was stopped with 2N NaOH (400 µL) and water (1 mL), and 4-hydroxyquinoline formation (Sigma-Aldrich, St. Louis, MO, USA) was measured spectrofluorometrically (λex/em = 310/400). IC50 values were determined using GraphPad Prism software (version 10, San Diego, CA, USA), and selectivity indices (*Si*) were calculated as Si of MAO-A = IC_50_ MAO-B/IC_50_ MAO-A and *Si* of MAO-B = IC_50_ MAO-A/IC_50_ MAO-B [22].

### 4.3. In Vivo Studies

#### 4.3.1. Animals, Husbandry, and Ethics Approval

Five-week-old male ICR mice (15–20 g) were obtained from Nomura Siam International (Bangkok, Thailand) and acclimatized for seven days prior to experimentation. The study protocol adhered to the Guiding Principle for the Care and Use of Animals (NIH Publications #8-23, revised 2011) and was approved by the Animal Ethics Committee of Khon Kaen University (approval No. IACUC-KKU-121/64), complying with ARRIVE guideline 2.0. Mice were housed on wood chip bedding in stainless cages with ad libitum access to water and food, maintained under a 12 h light–dark cycle (lights on 06:00–18:00 h), controlled temperature (22 ± 2 °C), and constant humidity (45% ± 2%) at the Laboratory Animal Unit, Faculty of Pharmaceutical Sciences, Khon Kaen University, Khon Kaen, Thailand. Behavioral experiments were conducted between 07:00 and 17:00 h.

#### 4.3.2. Chronic Mild Stress (CMS)-Induced Depression Procedure

**Figure 11:**
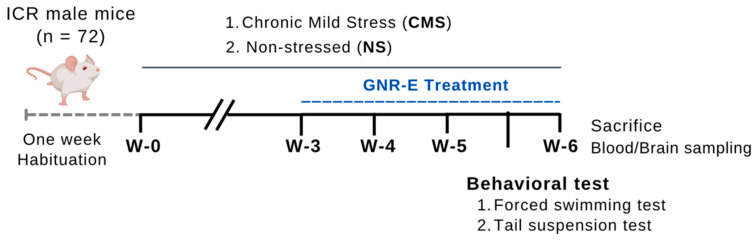
Schematic drawing of the chronic mild stress (CMS) procedure.

The CMS procedure, adapted from Willner [4], was applied to experimental mice over 48 days. Seventy-two mice were randomly assigned to six groups: a non-stress control group and four CMS-exposed groups for 6 weeks. Stressors included a tilted cage at 45° (12 h), restricted food access (five micro pellets, 1 h), empty bottle exposure (3 h), wet cage (200 mL water in 100 g sawdust bedding, 21 h), reversed light–dark cycle (36 h), intermittent sound (3 and 5 h), paired caging (2 h), and food/water deprivation (18 h), applied randomly weekly and repeated over 6 weeks (Figure 11). The non-stressed group was maintained under standard conditions [23].

#### 4.3.3. Drug Administration

Mice were randomly divided into six groups (n = 15). The non-stress group received 0.5% sodium carboxymethylcellulose (SCMC; 1 mL/kg, p.o.), while the CMS group received 0.5% SCMC (1 mL/kg, p.o.). The CMS + imipramine (IMP) group was administered imipramine hydrochloride (20 mg/kg, p.o.), and the CMS + GNR-E groups received GNR-E at 50, 150, and 450 mg/kg (p.o.), respectively. All treatments were given daily for 3 weeks, starting on day 21, at 8:00 a.m., one hour before behavioral testing.

#### 4.3.4. Sucrose Consumption Test (SCT)

After acclimatization, we conducted the sucrose consumption training prior to the baseline assessment. In week 0, all mice first underwent a 48 h food and water deprivation, after which mice were allowed to consume a 2% (*w*/*v*) sucrose solution. Following this initial training, the mice were subsequently deprived of food and water for 18 h and then given access to the 2% sucrose solution for 1 h, three consecutive days. The amount consumed each day was recorded and used to group the mice and establish the baseline sucrose consumption before initiating the chronic stress procedure. During the subsequent experimental period (week 1–6), sucrose consumption was measured weekly using the same deprivation and testing conditions under chronic stress. The weight of each mouse and the amount of sucrose solution consumed were recorded and calculated as the amount of sucrose intake g/kg body weight). The depressive mice showed a reduction in sucrose consumption [24].

#### 4.3.5. Tail Suspension Test (TST)

The tail suspension test (TST) was employed to assess antidepressant-like behavioral effects [25]. Mice were suspended by their tails using black adhesive tape, approximately 5 cm from the tip, with their heads positioned 40 cm above the floor. The TST apparatus (35 cm length, 40 cm width, 50 cm height) was set in a quiet, dark room. Immobility time was recorded for 6 min, starting 4 min after suspension, with increased immobility time serving as an index of depression.

#### 4.3.6. Forced Swimming Test (FST)

The forced swimming test (FST) was used to evaluate antidepressant-like behavioral effects, assessing active (swimming, climbing) or passive (immobility) responses in mice forced to swim in an inescapable environment [26]. Mice were individually placed in a transparent Plexiglas cylinder (20 cm diameter, 30 cm height) filled with water to 18 cm depth. In a pre-test session, mice swam for 15 min without observation. Twenty-four hours later, a 5 min test session was conducted under the same conditions. Immobility time was recorded when mice ceased struggling, floating motionless with minimal movement to keep their noses above the water surface.

### 4.4. Neurochemical Studies

#### 4.4.1. Blood Collection and Brain Preparation

Following behavioral tests, mice were anesthetized with thiopental sodium (Nembutal^®^, Ceva Sante Animal, Libourne, France) at 60 mg/kg (i.p.) and decapitated. Blood and brain samples were collected immediately. Blood was centrifuged at 3000 rpm at 21 °C for 20 min to obtain serum for ELISA analysis. The frontal cortex (FC) and hippocampus (HP) were dissected from the brain for serotonin (5-HT), norepinephrine (NE) level assessments, and RT-qPCR analyses.

#### 4.4.2. Brain Extraction for Estimation of Neurotransmitter Level

Frontal cortex (FC) and hippocampus (HP) tissues were homogenized with 1 mL HCl-butanol (0.85 mL 37% HCl in 1 L n-butanol for spectroscopy) for 1 min using a glass homogenizer. Samples were centrifuged at 2000 rpm at 4 °C for 10 min. An aliquot of supernatant (0.08 mL) was transferred to an Eppendorf tube with 0.2 mL heptane and 0.025 mL HCl (0.1 M). After 10 min, the mixture was vigorously agitated and centrifuged under the same conditions. The upper organic layer was discarded, and the aqueous phase was used to measure 5-HT and norepinephrine levels [27].

#### 4.4.3. Plasma Corticosterone (CORT) Assay

Corticosterone levels were measured using an enzyme-linked immunosorbent assay (ELISA) kit (ab108821, Abcam Ltd., Cambridge, Cambridgeshire, UK) following the manufacturer’s instructions. To control for circadian variation, blood samples were collected at zeitgeber time (ZT) 4–6, corresponding to 10:00–12:00 h under a 12:12 h light–dark cycle (lights on at 06:00 h). All mice were handled gently and consistently by the same trained experimenter for at least 3 consecutive days before sampling to minimize handling stress. During sample collection, animals were rapidly anesthetized, and blood was obtained within 2–3 min to avoid stress-induced CORT elevation. Serum was separated by centrifugation (3000× *g*, 10 min, 4 °C) and stored at −80 °C until analysis.

#### 4.4.4. Determination of Serotonin (5-HT) Levels

Serotonin levels in mouse brain extracts were quantified using a modified fluorometric method [28].A 0.2 mL brain extract sample was mixed with 0.25 mL O-phthaldialdehyde (Sigma-Aldrich, St. Louis, MO, USA) and heated to 100 °C for 10 min to form a fluorophore. Levels were measured spectrofluorometrically at 340 nm (excitation) and 470 nm (emission) after cooling to room temperature. Concentrations were determined against a calibration curve (0, 25, 50, 100, 200, 300, 400 µg/mL) using the equation Y = AX + B, expressed as µg/mL.

#### 4.4.5. Determination of Norepinephrine (NE) Levels

Brain supernatant (0.2 mL) was combined with 0.1 mL sodium acetate buffer (pH 6.9), 0.05 mL HCl (0.4 M), and 0.1 mL iodine solution (0.1 M in ethanol) for oxidation. After 2 min, 0.1 mL Na_2_SO_3_ solution was added to stop the reaction, followed by 0.1 mL acetic acid (10 M) after a 1.5 min incubation. The mixture was heated to 100 °C for 6 min, then cooled to ambient temperature. Excitation and emission spectra were recorded at 395 nm and 485 nm, respectively, using a calibrated spectrofluorometer. Norepinephrine concentrations were calculated from a standard curve (0, 50, 100, 140, 220, 280, 300 µg/mL) prepared in distilled water and butanol/HCl (1:2 ratio) [29].

#### 4.4.6. Quantitative Real-Time Polymerase Chain Reaction (QPCR)

Total RNA was extracted from frontal cortex (FC) and hippocampus (HP) using TRLzol^®^ reagent (Thermo Fisher Scientific Inc., San Jose, CA, USA), with purity and quantity assessed via UV spectrophotometry. Complementary DNA (cDNA) was synthesized using 10 mM dNTP, 50 µM oligo (dT)12-13 primer, and SuperScript^®^ III reverse transcriptase (200 U/µL) in a FlexCycler2 PCR Thermal Cycler (Analytik Jena, Thuringia, Germany). Single-stranded DNA was annealed to gene-specific primers (Pacific Science, Bangkok, Thailand) at optimized temperatures, followed by extension. Double-stranded DNA was quantified using SYBR Green master mix (Thermo Fisher Scientific Inc., San Jose, CA, USA) in a 20 µL reaction containing 2X SYBR Green PCR Master Mix, 10 nM of each primer, and 5 µL cDNA. Primers were synthesized by Macrogen (Seoul, Republic of Korea) as detailed in Table 3. Melting curve analysis was conducted post-amplification. Glyceraldehyde 3-phosphate dehydrogenase (*GAPDH*) mRNA served as the housekeeping gene, and fold differences were calculated using the 2^−∆∆CT^ method, incorporating standard deviation into the fold-difference calculation [30].

## 5. Statistical Analysis

Data were expressed as mean ± standard error of the mean (SEM). One-way ANOVA followed by Tukey’s Post Hoc test was performed using SigmaStat^®^ version 3.5 (SYSTAT Software Inc., San Jose, CA, USA). To control type I error, Tukey’s correction was applied for multiple comparisons. Statistical power (1 − β) and effect sizes (partial η^2^ and Cohen’s f) were calculated using G*Power version 3.1 (Heinrich Heine University, Düsseldorf, Germany). Results summarized in Supplementary Tables S18–S49 indicated adequate power (1 − β > 0.8) for all key endpoints (behavioral, n = 12; molecular, n = 6).

## 6. Conclusions

This study demonstrates the antidepressant-like effects of GNR-E in a chronic mild stress (CMS) mouse model of depression. Oral administration of GNR-E (50, 150, and 450 mg/kg/day) and imipramine (20 mg/kg/day) significantly improved depressive-like behaviors. The therapeutic effects of GNR-E in this preclinical model are likely mediated by enhanced serotonergic (5-HT) and noradrenergic (NE) neurotransmission, normalization of hypothalamic–pituitary–adrenal (HPA) axis hyperactivity (evidenced by reduced serum corticosterone levels), and restored mRNA expression of *SGK-1* and glucocorticoid receptor (*GR*) in the frontal cortex (FC) and hippocampus (HP). Additionally, GNR-E extract exhibited a stronger inhibitory activity toward monoamine oxidase-B (MAO-B) than monoamine oxidase-A (MAO-A), suggesting its potential relevance to dopaminergic regulation, cognitive impairment and Parkinson’s disease.

These findings provide preclinical evidence supporting the potential of GNR-E as an antidepressant-like agent, but it should be emphasized that translation to clinical use in humans requires further investigation, including dose optimization, comprehensive safety assessment, and controlled clinical trials. Thus, GNR-E emerges as a potential candidate for advancement as a future antidepressant.

## Figures and Tables

**Table 1:** Inhibitory effects of GNR-E, clorgyline, and deprenyl on monoamine oxidase A and B activities.

Sample	IC_50_ (µg/mL)		*Ki* (µg/mL)		Si	
	MAO-A	MAO-B	MAO-A	MAO-B	MAO-A	MAO-B
GNR-E	8.81 ± 0.02	3.60 ± 0.04	2.32	1.55	1.50	0.67
Clorgyline *	0.01 ± 0.001 µM	0.02 ± 0.002 µM	0.001	0.07	0.14	7.0
Deprenyl **	8.48 ± 0.14 µM	0.10 ± 0.02 µM	2.23	0.05	48.57	0.02

* Clorgyline: selective for MAO-A; ** Deprenyl: selective for MAO-B; *Ki*: Enzyme-inhibitor dissociation constant; Si: selectivity index.

**Table 2:** Validation results of the HPLC method for quantifying standard compounds isolated from GNR-E.

Parameters		Standard Compounds				
		1	2	3	4	5
LOD	Concentration (µg/mL)	0.5	0.5	0.1	0.5	0.2
	S/N	3.08 ± 0.06	3.09 ± 0.07	3.05 ± 0.02	3.09 ± 0.06	3.08 ± 0.11
LOQ	Concentration (µg/mL)	1.0	5.0	0.5	1.0	1.0
	S/N	10.77 ± 0.66	9.81 ± 0.55	10.81 ± 0.69	10.86 ± 0.83	10.78 ± 0.92
Linearity	Range (µg/mL)	1–20	5–30	1–20	0.5–15	5–13
	Equation	y = 7.2427x − 6.8218	y = 0.6813x − 0.2263	y = 19.705x − 5.7932	y = 7.4318x − 1.3177	y = 3.0641x − 10.690
	(R^2^)	0.9976	0.9945	0.9918	0.9970	0.9939
Precision (%RSD)	Repeatability	0.18–1.64%	0.07–1.13%	0.02–1.19%	0.03–1.75%	0.03–1.93%
	Intermediate precision	0.07–1.62%	0.53–3.02%	0.35–1.37%	0.52–1.36%	0.18–1.59%
Accuracy (%recovery)	Low concentration	98.73 ± 0.05	108.34 ± 0.79	101.51 ± 0.25	100.47 ± 0.08	96.19 ± 0.43
	Medium concentration	10.29 ± 0.45	95.25 ± 0.09	108.22 ± 0.58	104.33 ± 0.01	102.82 ± 0.83
	High concentration	98.35 ± 0.13	102.71 ± 0.75	98.99 ± 0.06	96.33 ± 0.39	98.78 ± 0.08
Robustness	0.9 (mL/min) (% RSD)	0.38	0.45	0.41	0.39	0.31
	1.1 (mL/min) (% RSD)	0.17	0.21	0.02	0.26	0.26
GNR-E (mg/g extract)		0.070 ± 0.004	0.377 ± 0.069	0.101 ± 0.002	0.076 ± 0.006	1.916 ± 0.01

**Table 3:** The primer sequences used for qPCR were analyzed.

Genes	Forward (5′-3′)	Reward (5′-3′)	BP	Accession No.
*SERT*	CTCATCTTCACATTATCTACTTCAG	CTCACCAGCAGGACAGAAAG	113	NM_010484.2
*5HT1A*	CTGTGACCTGTTTATCGCCCTG	GTAGTCTATAGGGTCGGTGATTGC	109	NM_008308.5
*5HT1B*	ACATCCTCGGTCACCTCCATTA	CCCTAGCGGCCATGAGTTTC	136	NM_000863.3
*5HT2A*	CAGCGGTCCATCCACAGAG	CCACATTACAACAAACAGAAAGAACAC	123	NM_172812.3
*5HT2B*	AAGCCAATTCAGGCCAATC	GGGCACCACATAAGCAGAAA	542	NM_008311.3
*5HT2C*	GGTCCTTCGTGGCATTCTTCATC	CGCAGTTCCTCCTCGGTGTG	121	NM_001411391.1
*5HT6*	CCTGGTGTCGCTCTTCACG	GGCATCACCACCAATCCC	51	NM_001377096.1
*5HT7*	GTTAGTGTCACGGACCTCAT	ATCATTTTGGCCATACATTT	255	NM_001360300.1
*NET*	TGCACGAGAGCAGTGGGAT	CGACCATCAGGCAGAGCAG	69	NM_009209.3
*α2A*	GTGACACTGACGCTGGTTTG	CCAGTAACCCATAACCTCGTTG	204	NM_007417.5
*α2C*	CTGTGGTGGGTTTCCTCATCG	ACTTGCCCGAAGTACCAGTAG	199	NM_007418.3
*GR*	CACTAATCCTCTCCATCCTAC	AATGTCTGCTGCCTTCTG	479	NM_008173.4
*SGK-1*	GGGTGCCAAGGATGACTTTA	CTCGGTAAACTCGGGATAGA	154	NM_011361.3

## Data Availability

The original contributions presented in this study are included in the article/supplementary material. Further inquiries can be directed to the corresponding authors.

## Supplementary Materials

The following supporting information can be downloaded at: https://www.mdpi.com/article/10.3390/plants14233651/s1, Table S1: One-way ANOVA results for the sucrose preference test. Table S2: One-way ANOVA results for the forced swim test (FST). Table S3: One-way ANOVA results for the tail suspension test (TST). Table S4: One-way ANOVA results for CMS-induced serum corticosterone (CORT) levels. Table S5: One-way ANOVA results for serotonin (5-HT) levels in the frontal cortex. Table S6: One-way ANOVA results for norepinephrine (NE) levels in the frontal cortex. Table S7: One-way ANOVA results for *SERT* gene expression in the frontal cortex and hippocampus. Table S8: One-way ANOVA results for *5HT1A* gene expression in the frontal cortex and hippocampus. Table S9: One-way ANOVA results for *5HT1B* gene expression in the frontal cortex and hippocampus. Table S10: One-way ANOVA results for *5HT2A* gene expression in the frontal cortex and hippocampus. Table S11: One-way ANOVA results for *5HT2C* gene expression in the frontal cortex and hippocampus. Table S12: One-way ANOVA results for *5HT7* gene expression in the frontal cortex and hippocampus. Table S13: One-way ANOVA results for *NET* gene expression in the frontal cortex and hippocampus. Table S14: One-way ANOVA results for *α2A* gene expression in the frontal cortex and hippocampus. Table S15: One-way ANOVA results for *α2C* gene expression in the frontal cortex and hippocampus. Table S16: One-way ANOVA results for *GR* gene expression in the frontal cortex and hippocampus. Table S17: One-way ANOVA results for *SGK-1* gene expression in the frontal cortex and hippocampus. Table S18. FDR and Bonferroni results for the sucrose preference test. Tables S19 and S20. FDR and Bonferroni results for the FST and TST. Table S21. FDR and Bonferroni results for the CORT levels. Tables S22–S25. FDR and Bonferroni results for the 5-HT and NE levels in frontal cortex and hippocampus. Tables S26–S47. FDR and Bonferroni results for the *SERT*, *5HT1A*, *5HT1B*, *5HT2A*, *5HT2C*, and *5HT7*, *NET*, *2A*, *2C GR* and *SGK-1* on biomarkers mRNA expression in frontal cortex and hippocampus. Tables S48 and S49. Effect size (partial η^2^), Cohen’s f, and achieved statistical power (1 − β) for monoaminergic, receptor, and glucocorticoid-related markers in the frontal cortex and hippocampus.
