# Supplementary material for: Antidepressant-like Effects of Garcinia nigrolineata Resin Extract in a Chronic Mild Stress Mouse Model: Modulation of Monoaminergic and HPA-Axis Pathways
Source: Plants (Basel). 2025 Nov 30;14(23):3651. doi: 10.3390/plants14233651 (PMC12693764; doi:10.3390/plants14233651)
Supplement: Supplementary file 1 [file plants-14-03651-s001.zip › plants-3973351-supplementary.pdf]

Supplementary Materials

Antidepressant-like Effects of *Garcinia nigrolineata* Resin Extract in a Chronic Mild Stress Mouse Model: Modulation of Monoaminergic and HPA-Axis Pathways

Yutthana Chotritthirong [1, 2], Yaowared Sumanont [2], Supawadee Daodee [2], Abdulwaris Mading [1,2], Chantana Boonyarat [2,] Charinya Khamphukdee [3], Decha Kumla [4], Juthamart Maneenet [5], Kinzo Matsumoto [6], Anake Kijjoa [7], Suresh Awale [5, *] and Orawan Monthakantirat [2,*]

[1] Graduate School of Faculty of Pharmaceutical Sciences, Khon Kaen University, Khon Kaen 40002, Thailand.

[2] Division of Pharmaceutical Chemistry, Faculty of Pharmaceutical Sciences, Khon Kaen University, Khon Kaen 40002, Thailand.

[3] Division of Pharmacognosy and Toxicology, Faculty of Pharmaceutical Sciences, Khon Kaen University, Khon Kaen 40002, Thailand.

[4] Faculty of Pharmaceutical Sciences, Burapha University, 169 Long Had Bangsaen Road, Chonburi 20131, Thailand.

[5] Natural Drug Discovery Laboratory, Institute of Natural Medicine, University of Toyama, 2630 Sugitani, Toyama 930-0145, Japan.

[6] Graduate School of Pharmaceutical Sciences, Daiichi University of Pharmacy, Fukuoka 815-8511, Japan.

[7] ICBAS-Instituto de Ciências Biomédicas Abel Salazar and CIIMAR, Universidade do Porto, Rua de Jorge Viterbo Ferreira 228, 4050-313 Porto, Portugal.

* Correspondence: suresh@inm.u-toyama.ac.jp (S.A.); oramon@kku.ac.th (O.M.); Tel.: +66-81-3404677

Table of Contents

Page

1. Statistical analysis of the effect of *Garcinia nigrolineata* resin extract (GNR-E) on CMS-induced anhedonia behavior using the sucrose preference test

   Table S1. One-way analysis of variance (ANOVA) test of sucrose preference test (Week 6)... 4

2. Statistical analysis of the effect of *Garcinia nigrolineata* resin extract (GNR-E) on CMS-induced hopeless behavior using forced swimming test (FST) and tail suspension test (TST)

   Table S2. One-way analysis of variance (ANOVA) test of FST………………...……………. 4

   Table S3. One-way analysis of variance (ANOVA) test of TST……...…………………………. 4

3. Statistical analysis of the effect of *Garcinia nigrolineata* resin extract (GNR-E) on CMS induced hypersecretion of the serum corticosterone levels

   Table S4. One-way analysis of variance (ANOVA) test of CMS-induced hypersecretion of the serum corticosterone (CORT) levels.. 5

4. Statistical analysis of the effect of *Garcinia nigrolineata* resin extract (GNR-E) on CMS-induced changes in serotonin (5-HT) and norepinephrine (NE) levels in frontal cortex and hippocampus

   Table S5. One-way analysis of variance (ANOVA) test of serotonin (5-HT) levels.................. 5

   Table S6. One-way analysis of variance (ANOVA) test of norepinephrine (NE) levels........... 6

5. Statistical analysis of the effect of *Garcinia nigrolineata* resin extract (GNR-E) on CMS-induced changes in serotonin transporter (SERT), serotonin receptor (5HT1A, 5HT1B, 5HT2A, 5HT2C, and 5HT7), norepinephrine transporter (NET), norepinephrine Receptor (α2A, and α2C) and HPA-axis system (GR and SGK-1) biomarkers mRNA expression in frontal cortex and hippocampus

   Table S7. One-way analysis of variance (ANOVA) test of SERT gene expression in the frontal cortex and hippocampus……………………………………………………………... 7

   Table S8. One-way analysis of variance (ANOVA) test of 5HT1A gene expression in the frontal cortex and hippocampus……………………………………………………………... 8

   Table S9. One-way analysis of variance (ANOVA) test of 5HT1B gene expression in the frontal cortex and hippocampus……………………………………………………………... 9

   Table S10. One-way analysis of variance (ANOVA) test of 5HT2A gene expression in the frontal cortex and hippocampus……………………………………………………………... 10

   Table S11. One-way analysis of variance (ANOVA) test of 5HT2C gene expression in the frontal cortex and hippocampus……………………………………………………………... 11

Table of Contents (Cont.)

Page

Table S12. One-way analysis of variance (ANOVA) test of 5HT7 gene expression in the frontal cortex and hippocampus……………………………………………………………... 12

Table S13. One-way analysis of variance (ANOVA) test of NET gene expression in the frontal cortex and hippocampus…………………………………………………………….. 13

Table S14. One-way analysis of variance (ANOVA) test of α2A gene expression in the frontal cortex and hippocampus……………………………………………………………... 14

Table S15. One-way analysis of variance (ANOVA) test of α2C gene expression in the frontal cortex and hippocampus……………………………………………………………... 15

Table S16. One-way analysis of variance (ANOVA) test of GR gene expression in the frontal cortex and hippocampus……………………………………………………………... 16

Table S17. One-way analysis of variance (ANOVA) test of SGK-1 gene expression in the frontal cortex and hippocampus……………………………………………………………... 17

Table S18. FDR and Bonferroni analysis of the effect of *Garcinia nigrolineata* resin extract (GNR-E) on CMS-induced anhedonia behavior using the sucrose preference test 19

Table S19. FDR and Bonferroni analysis of the effect of *Garcinia nigrolineata* resin extract (GNR-E) on CMS-induced hopeless behavior using forced swimming test (FST). 20

Table S20. FDR and Bonferroni analysis the effect of *Garcinia nigrolineata* resin extract (GNR-E) on CMS-induced hopeless behavior using tail suspension test (TST). 20

Table S21. FDR and Bonferroni analysis of CMS-induced hypersecretion of the serum corticosterone (CORT) levels……………………………………………………………………. 20

Table S22-25. FDR and Bonferroni analysis of the effect of *Garcinia nigrolineata* resin extract (GNR-E) on CMS-induced changes in serotonin (5-HT) and norepinephrine (NE) levels in frontal cortex and hippocampus. ………………………………………………………………. 21

Table S26-47. FDR and Bonferroni analysis of the effect of *Garcinia nigrolineata* resin extract (GNR-E) on CMS-induced changes in serotonin transporter (SERT), serotonin receptor (5HT1A, 5HT1B, 5HT2A, 5HT2C, and 5HT7), norepinephrine transporter (NET), norepinephrine Receptor (2A, and 2C) and HPA-axis system (GR and SGK-1) biomarkers mRNA expression in frontal cortex and hippocampus. ……………………………………… 22-30

Table 48. Effect size (partial η^2), Cohen's f, and achieved statistical power ($1-\beta$) for monoaminergic, receptor, and glucocorticoid-related markers in the frontal cortex and hippocampus. 31

Table 49. Effect size (partial η^2) of biological relevance in Frontal Cortex and Hippocampus. 32

1. Statistical analysis of the effect of *Garcinia nigrolineata* resin extract (GNR-E) on CMS-induced anhedonia behavior using the sucrose preference test.

Table S1. One-way analysis of variance (ANOVA) test of the sucrose consumption test (Week 6).

Group comparison	ANOVA followed by Tukey's post hoc test	
	P	*F* ($DF_{between\ group}$, $DF_{residual}$
All group	<0.0001	F(4,55)=12.932
non-stress group vs. CMS + vehicle group	<0.001	
CMS + vehicle group vs. CMS + IMP20 group	<0.001	
CMS + vehicle group vs. CMS + GNRS 50 group	<0.001	
CMS + vehicle group vs. CMS + GNRS 150 group	<0.001	
CMS + vehicle group vs. CMS + GNRS 450 group	<0.001	

2. Statistical analysis of the effect of *Garcinia nigrolineata* resin extract (GNR-E) on CMS-induced hopeless behavior using forced swimming test (FST) and tail suspension test (TST).

Table S2. One-way analysis of variance (ANOVA) test of FST.

Group comparison	ANOVA followed by Tukey's post hoc test	
	P	*F* ($DF_{between\ group}$, $DF_{residual}$
All group	<0.001	F(4,55)=53.063
non-stress group vs. CMS + vehicle group	<0.001	
CMS + vehicle group vs. CMS + IMP20 group	<0.001	
CMS + vehicle group vs. CMS + GNRS 50 group	<0.001	
CMS + vehicle group vs. CMS + GNRS 150 group	<0.001	
CMS + vehicle group vs. CMS + GNRS 450 group	<0.001	

Table S3. One-way analysis of variance (ANOVA) test of TST.

Group comparison	ANOVA followed by Tukey's post hoc test	
	P	*F* ($DF_{between\ group}$, $DF_{residual}$
All group	<0.001	F(4,55)=53.063
non-stress group vs. CMS + vehicle group	<0.001	
CMS + vehicle group vs. CMS + IMP20 group	<0.001	
CMS + vehicle group vs. CMS + GNRS 50 group	<0.001	
CMS + vehicle group vs. CMS + GNRS 150 group	<0.001	
CMS + vehicle group vs. CMS + GNRS 450 group	<0.001	
CMS + GNRS 50 group vs. CMS + GNRS 450 group	0.05	

3. Statistical analysis of the effect of *Garcinia nigrolineata* resin extract (GNR-E) on CMS-induced hypersecretion of the serum corticosterone levels.

Table S4. One-way analysis of variance (ANOVA) test of CMS-induced hypersecretion of the serum corticosterone (CORT) levels.

Group comparison	ANOVA followed by Tukey's post hoc test	
	P	*F* ($DF_{between\ group}$, $DF_{residual}$
All group	<0.001	*F*(4,15)=284.816
non-stress group vs. CMS + vehicle group	<0.001	
CMS + vehicle group vs. CMS + IMP20 group	<0.001	
CMS + vehicle group vs. CMS + GNRS 50 group	<0.001	
CMS + vehicle group vs. CMS + GNRS 150 group	<0.001	
CMS + vehicle group vs. CMS + GNRS 450 group	<0.001	
CMS + GNRS 50 group vs. CMS + GNRS 450 group	<0.001	

4. Statistical analysis of the effect of *Garcinia nigrolineata* resin extract (GNR-E) on CMS-induced changes in serotonin (5-HT) and norepinephrine (NE) levels in frontal cortex and hippocampus.

Table S5. One-way analysis of variance (ANOVA) test of serotonin (5-HT) levels.

Group comparison	ANOVA followed by Tukey's post hoc test	
	P	*F* ($DF_{between\ group}$, $DF_{residual}$
Frontal cortex		
All group	0.004	*F*(4,15)=23.154
non-stress group vs. CMS + vehicle group	<0.001	
CMS + vehicle group vs. CMS + IMP20 group	0.018	
CMS + vehicle group vs. CMS + GNRS 50 group	0.168	
CMS + vehicle group vs. CMS + GNRS 150 group	0.207	
CMS + vehicle group vs. CMS + GNRS 450 group	0.002	
Hippocampus		
All group	<0.001	*F*(4,15)=22.868
non-stress group vs. CMS + vehicle group	<0.001	
CMS + vehicle group vs. CMS + IMP20 group	<0.001	
CMS + vehicle group vs. CMS + GNRS 50 group	0.203	
CMS + vehicle group vs. CMS + GNRS 150 group	0.012	
CMS + vehicle group vs. CMS + GNRS 450 group	<0.001	
CMS + GNRS 50 group vs. CMS + GNRS 450 group	<0.001	

Table S6. One-way analysis of variance (ANOVA) test of norepinephrine (NE) levels.

Group comparison	ANOVA followed by Tukey's post hoc test	
	P	*F* ($DF_{between\ group}$, $DF_{residual}$
Frontal cortex		
All group	<0.001	$F(4,17)=65.963$
non-stress group vs. CMS + vehicle group	<0.001	
CMS + vehicle group vs. CMS + IMP20 group	<0.001	
CMS + vehicle group vs. CMS + GNRS 50 group	0.069	
CMS + vehicle group vs. CMS + GNRS 150 group	<0.001	
CMS + vehicle group vs. CMS + GNRS 450 group	<0.001	
CMS + GNRS 50 group vs. CMS + GNRS 450 group	<0.001	
CMS + GNRS 150 group vs. CMS + GNRS 450 group	0.026	
Hippocampus		
All group	<0.001	$F(4,17)=51.154$
non-stress group vs. CMS + vehicle group	<0.001	
CMS + vehicle group vs. CMS + IMP20 group	<0.001	
CMS + vehicle group vs. CMS + GNRS 50 group	0.998	
CMS + vehicle group vs. CMS + GNRS 150 group	0.158	
CMS + vehicle group vs. CMS + GNRS 450 group	<0.001	
CMS + GNRS 50 group vs. CMS + GNRS 450 group	<0.001	
CMS + GNRS 150 group vs. CMS + GNRS 450 group	0.017	

5. Statistical analysis of the effect of *Garcinia nigrolineata* resin extract (GNR-E) on CMS-induced changes in serotonin transporter (SERT), serotonin receptor (5HT1A, 5HT1B, 5HT2A, 5HT2C, and 5HT7), norepinephrine transporter (NET), norepinephrine Receptor (α2A, and α2C) and HPA-axis system (GR and SGK-1) biomarkers mRNA expression in frontal cortex and hippocampus.

Table S7. One-way analysis of variance (ANOVA) test of SERT gene expression in the frontal cortex and hippocampus.

Group comparison	ANOVA followed by Tukey's post hoc test	
	P	F ($DF_{between\ group}$, $DF_{residual}$
Frontal cortex		
All group	<0.001	$F(4,25)=14.189$
non-stress group vs. CMS + vehicle group	<0.001	
CMS + vehicle group vs. CMS + IMP20 group	<0.001	
CMS + vehicle group vs. CMS + GNRS 50 group	0.930	
CMS + vehicle group vs. CMS + GNRS 150 group	0.404	
CMS + vehicle group vs. CMS + GNRS 450 group	<0.001	
CMS + GNRS 50 group vs. CMS + GNRS 450 group	No sig.	
CMS + GNRS 150 group vs. CMS + GNRS 450 group	No sig.	
Hippocampus		
All group	<0.001	$F(4,25)=19.749$
non-stress group vs. CMS + vehicle group	<0.001	
CMS + vehicle group vs. CMS + IMP20 group	<0.001	
CMS + vehicle group vs. CMS + GNRS 50 group	<0.001	
CMS + vehicle group vs. CMS + GNRS 150 group	<0.001	
CMS + vehicle group vs. CMS + GNRS 450 group	<0.001	
CMS + GNRS 50 group vs. CMS + GNRS 450 group	No sig.	
CMS + GNRS 150 group vs. CMS + GNRS 450 group	No sig.	

Table S8. One-way analysis of variance (ANOVA) test of 5HT1A gene expression in the frontal cortex and hippocampus.

Group comparison	ANOVA followed by Tukey's post hoc test	
	P	*F* ($DF_{between\ group}$, $DF_{residual}$
Frontal cortex		
All group	<0.001	F(4,25)=12.755
non-stress group vs. CMS + vehicle group	<0.001	
CMS + vehicle group vs. CMS + IMP20 group	<0.001	
CMS + vehicle group vs. CMS + GNRS 50 group	0.117	
CMS + vehicle group vs. CMS + GNRS 150 group	0.005	
CMS + vehicle group vs. CMS + GNRS 450 group	<0.001	
CMS + GNRS 50 group vs. CMS + GNRS 450 group	0.033	
CMS + GNRS 150 group vs. CMS + GNRS 450 group	No sig.	
Hippocampus		
All group	0.003	F(4,24)=5.259
non-stress group vs. CMS + vehicle group	0.002	
CMS + vehicle group vs. CMS + IMP20 group	0.019	
CMS + vehicle group vs. CMS + GNRS 50 group	0.402	
CMS + vehicle group vs. CMS + GNRS 150 group	0.095	
CMS + vehicle group vs. CMS + GNRS 450 group	0.003	
CMS + GNRS 50 group vs. CMS + GNRS 450 group	No sig.	
CMS + GNRS 150 group vs. CMS + GNRS 450 group	No sig.	

Table S9. One-way analysis of variance (ANOVA) test of 5HT1B gene expression in the frontal cortex and hippocampus.

Group comparison	ANOVA followed by Tukey's post hoc test	
	P	*F* ($DF_{between\ group}$, $DF_{residual}$
Frontal cortex		
All group	<0.001	$F(4,25)=16.639$
non-stress group vs. CMS + vehicle group	<0.001	
CMS + vehicle group vs. CMS + IMP20 group	<0.001	
CMS + vehicle group vs. CMS + GNRS 50 group	<0.001	
CMS + vehicle group vs. CMS + GNRS 150 group	0.001	
CMS + vehicle group vs. CMS + GNRS 450 group	<0.001	
CMS + GNRS 50 group vs. CMS + GNRS 450 group	No.sig	
CMS + GNRS 150 group vs. CMS + GNRS 450 group	No sig.	
Hippocampus		
All group	<0.001	$F(4,25)=8.883$
non-stress group vs. CMS + vehicle group	<0.001	
CMS + vehicle group vs. CMS + IMP20 group	<0.001	
CMS + vehicle group vs. CMS + GNRS 50 group	0.687	
CMS + vehicle group vs. CMS + GNRS 150 group	0.012	
CMS + vehicle group vs. CMS + GNRS 450 group	0.001	
CMS + GNRS 50 group vs. CMS + GNRS 450 group	0.032	
CMS + GNRS 150 group vs. CMS + GNRS 450 group	0.193	

Table S10. One-way analysis of variance (ANOVA) test of 5HT2A gene expression in the frontal cortex and hippocampus.

Group comparison	ANOVA followed by Tukey's post hoc test	
	P	*F* (DF$_{between\ group}$, DF$_{residual}$
Frontal cortex		
All group	0.001	*F*(4,25)=6.323
non-stress group vs. CMS + vehicle group	0.003	
CMS + vehicle group vs. CMS + IMP20 group	0.007	
CMS + vehicle group vs. CMS + GNRS 50 group	0.977	
CMS + vehicle group vs. CMS + GNRS 150 grop	0.071	
CMS + vehicle group vs. CMS + GNRS 450 group	0.008	
CMS + GNRS 50 group vs. CMS + GNRS 450 group	0.032	
CMS + GNRS 150 group vs. CMS + GNRS 450 group	No sig.	
Hippocampus		
All group	<0.001	*F*(4,15)=7.986
non-stress group vs. CMS + vehicle group	<0.001	
CMS + vehicle group vs. CMS + IMP20 group	<0.001	
CMS + vehicle group vs. CMS + GNRS 50 group	0.203	
CMS + vehicle group vs. CMS + GNRS 150 group	<0.001	
CMS + vehicle group vs. CMS + GNRS 450 group	<0.001	
CMS + GNRS 50 group vs. CMS + GNRS 450 group	No sig.	
CMS + GNRS 150 group vs. CMS + GNRS 450 group	No sig.	

Table S11. One-way analysis of variance (ANOVA) test of 5HT2C gene expression in the frontal cortex and hippocampus.

Group comparison	ANOVA followed by Tukey's post hoc test	
	P	*F* ($DF_{between\ group}$, $DF_{residual}$
Frontal cortex		
All group	<0.001	*F*(4,25)=17.254
non-stress group vs. CMS + vehicle group	<0.001	
CMS + vehicle group vs. CMS + IMP20 group	<0.001	
CMS + vehicle group vs. CMS + GNRS 50 group	<0.001	
CMS + vehicle group vs. CMS + GNRS 150 group	<0.001	
CMS + vehicle group vs. CMS + GNRS 450 group	<0.001	
CMS + GNRS 50 group vs. CMS + GNRS 450 group	No sig.	
CMS + GNRS 150 group vs. CMS + GNRS 450 group	No sig.	
Hippocampus		
All group	<0.001	*F*(4,25)=15.549
non-stress group vs. CMS + vehicle group	<0.001	
CMS + vehicle group vs. CMS + IMP20 group	<0.001	
CMS + vehicle group vs. CMS + GNRS 50 group	0.093	
CMS + vehicle group vs. CMS + GNRS 150 group	0.006	
CMS + vehicle group vs. CMS + GNRS 450 group	<0.001	
CMS + GNRS 50 group vs. CMS + GNRS 450 group	0.006	
CMS + GNRS 150 group vs. CMS + GNRS 450 group	No sig.	

Table S12. One-way analysis of variance (ANOVA) test of 5HT7 gene expression in the frontal cortex and hippocampus.

Group comparison	ANOVA followed by Tukey's post hoc test	
	P	F (DF$_{\text{between group}}$, DF$_{\text{residual}}$
Frontal cortex		
All group	<0.001	$F(4,25)=9.964$
non-stress group vs. CMS + vehicle group	<0.001	
CMS + vehicle group vs. CMS + IMP20 group	<0.001	
CMS + vehicle group vs. CMS + GNRS 50 group	0.003	
CMS + vehicle group vs. CMS + GNRS 150 group	0.037	
CMS + vehicle group vs. CMS + GNRS 450 group	<0.001	
CMS + GNRS 50 group vs. CMS + GNRS 450 group	No sig.	
CMS + GNRS 150 group vs. CMS + GNRS 450 group	No sig.	
Hippocampus		
All group	<0.001	$F(4,25)=8.285$
non-stress group vs. CMS + vehicle group	<0.001	
CMS + vehicle group vs. CMS + IMP20 group	<0.001	
CMS + vehicle group vs. CMS + GNRS 50 group	0.178	
CMS + vehicle group vs. CMS + GNRS 150 group	0.003	
CMS + vehicle group vs. CMS + GNRS 450 group	<0.001	
CMS + GNRS 50 group vs. CMS + GNRS 450 group	0.001	
CMS + GNRS 150 group vs. CMS + GNRS 450 group	No sig.	

Table S13. One-way analysis of variance (ANOVA) test of NET gene expression in the frontal cortex and hippocampus.

Group comparison	ANOVA followed by Tukey's post hoc test	
	P	*F* ($DF_{between\ group}$, $DF_{residual}$
Frontal cortex		
All group	<0.001	$F(4,25)=24.220$
non-stress group vs. CMS + vehicle group	<0.001	
CMS + vehicle group vs. CMS + IMP20 group	<0.001	
CMS + vehicle group vs. CMS + GNRS 50 group	0.984	
CMS + vehicle group vs. CMS + GNRS 150 group	0.093	
CMS + vehicle group vs. CMS + GNRS 450 group	<0.001	
CMS + GNRS 50 group vs. CMS + GNRS 450 group	<0.001	
CMS + GNRS 150 group vs. CMS + GNRS 450 group	No sig.	
Hippocampus		
All group	<0.001	$F(4,25)=18.640$
non-stress group vs. CMS + vehicle group	<0.001	
CMS + vehicle group vs. CMS + IMP20 group	<0.001	
CMS + vehicle group vs. CMS + GNRS 50 group	0.966	
CMS + vehicle group vs. CMS + GNRS 150 group	0.039	
CMS + vehicle group vs. CMS + GNRS 450 group	<0.001	
CMS + GNRS 50 group vs. CMS + GNRS 450 group	0.001	
CMS + GNRS 150 group vs. CMS + GNRS 450 group	No sig.	

Table S14. One-way analysis of variance (ANOVA) test of α2A gene expression in the frontal cortex and hippocampus.

Group comparison	ANOVA followed by Tukey's post hoc test	
	P	*F* ($DF_{between\ group}$, $DF_{residual}$
Frontal cortex		
All group	<0.001	$F(4,25)=27.652$
non-stress group vs. CMS + vehicle group	<0.001	
CMS + vehicle group vs. CMS + IMP20 group	<0.001	
CMS + vehicle group vs. CMS + GNRS 50 group	0.298	
CMS + vehicle group vs. CMS + GNRS 150 group	<0.001	
CMS + vehicle group vs. CMS + GNRS 450 group	<0.001	
CMS + GNRS 50 group vs. CMS + GNRS 450 group	No.sig	
CMS + GNRS 50 group vs. CMS + GNRS 450 group	<0.001	
Hippocampus		
All group	<0.001	$F(4,25)=87.633$
non-stress group vs. CMS + vehicle group	<0.001	
CMS + vehicle group vs. CMS + IMP20 group	<0.001	
CMS + vehicle group vs. CMS + GNRS 50 group	0.002	
CMS + vehicle group vs. CMS + GNRS 150 group	<0.001	
CMS + vehicle group vs. CMS + GNRS 450 group	<0.001	
CMS + GNRS 50 group vs. CMS + GNRS 450 group	<0.001	
CMS + GNRS 150 group vs. CMS + GNRS 450 group	<0.001	
CMS + GNRS 50 group vs. CMS + GNRS 150 group	<0.001	

Table S15. One-way analysis of variance (ANOVA) test of α2C gene expression in the frontal cortex and hippocampus.

Group comparison	ANOVA followed by Tukey's post hoc test	
	P	F ($DF_{between\ group}$, $DF_{residual}$
Frontal cortex		
All group	<0.001	$F(4,25)=50.751$
non-stress group vs. CMS + vehicle group	<0.001	
CMS + vehicle group vs. CMS + IMP20 group	<0.001	
CMS + vehicle group vs. CMS + GNRS 50 group	<0.001	
CMS + vehicle group vs. CMS + GNRS 150 group	<0.001	
CMS + vehicle group vs. CMS + GNRS 450 group	<0.001	
CMS + GNRS 50 group vs. CMS + GNRS 450 group	<0.001	
CMS + GNRS 150 group vs. CMS + GNRS 450 group	No sig.	
Hippocampus		
All group	<0.001	$F(4,12)=9.850$
non-stress group vs. CMS + vehicle group	<0.001	
CMS + vehicle group vs. CMS + IMP20 group	<0.001	
CMS + vehicle group vs. CMS + GNRS 50 group	0.951	
CMS + vehicle group vs. CMS + GNRS 150 group	0.384	
CMS + vehicle group vs. CMS + GNRS 450 group	0.013	
CMS + GNRS 50 group vs. CMS + GNRS 450 group	No sig.	
CMS + GNRS 150 group vs. CMS + GNRS 450 group	No sig.	

Table S16. One-way analysis of variance (ANOVA) test of GR gene expression in the frontal cortex and hippocampus.

Group comparison	ANOVA followed by Tukey's post hoc test	
	P	F ($DF_{between\ group}$, $DF_{residual}$
Frontal cortex		
All group	<0.001	$F(4,25)=55.901$
non-stress group vs. CMS + vehicle group	<0.001	
CMS + vehicle group vs. CMS + IMP20 group	<0.001	
CMS + vehicle group vs. CMS + GNRS 50 group	<0.001	
CMS + vehicle group vs. CMS + GNRS 150 group	<0.001	
CMS + vehicle group vs. CMS + GNRS 450 group	<0.001	
CMS + GNRS 50 group vs. CMS + GNRS 450 group	<0.001	
CMS + GNRS 150 group vs. CMS + GNRS 450 group	No.sig	
Hippocampus		
All group	<0.001	$F(4,25)=30.098$
non-stress group vs. CMS + vehicle group	<0.001	
CMS + vehicle group vs. CMS + IMP20 group	<0.001	
CMS + vehicle group vs. CMS + GNRS 50 group	0.012	
CMS + vehicle group vs. CMS + GNRS 150 group	<0.001	
CMS + vehicle group vs. CMS + GNRS 450 group	<0.001	
CMS + GNRS 50 group vs. CMS + GNRS 450 group	<0.001	
CMS + GNRS 150 group vs. CMS + GNRS 450 group	No sig.	

Table S17. One-way analysis of variance (ANOVA) test of SGK-1 gene expression in the frontal cortex and hippocampus.

Group comparison	ANOVA followed by Tukey's post hoc test	
	P	F (DF$_{between\ group}$, DF$_{residual}$
Frontal cortex		
All group	<0.001	F(4,25)=21.138
non-stress group vs. CMS + vehicle group	<0.001	
CMS + vehicle group vs. CMS + IMP20 group	<0.001	
CMS + vehicle group vs. CMS + GNRS 50 group	0.742	
CMS + vehicle group vs. CMS + GNRS 150 group	0.003	
CMS + vehicle group vs. CMS + GNRS 450 group	<0.001	
CMS + GNRS 50 group vs. CMS + GNRS 450 group	<0.001	
CMS + GNRS 150 group vs. CMS + GNRS 450 group	No sig.	
Hippocampus		
All group	<0.001	F(4,25)=25.837
non-stress group vs. CMS + vehicle group	<0.001	
CMS + vehicle group vs. CMS + IMP20 group	<0.001	
CMS + vehicle group vs. CMS + GNRS 50 group	0.131	
CMS + vehicle group vs. CMS + GNRS 150 group	<0.001	
CMS + vehicle group vs. CMS + GNRS 450 group	<0.001	
CMS + GNRS 50 group vs. CMS + GNRS 450 group	<0.001	
CMS + GNRS 150 group vs. CMS + GNRS 450 group	No sig.	
CMS + GNRS 50 group vs. CMS + GNRS 150 group	0.013	

FDR and Bonferron*i* analysis

Table S18. Statistical FDR and Bonferroni analysis of the effect of *Garcinia nigrolineata* resin extract (GNR-E) on CMS-induced anhedonia behavior using the sucrose consumption test

Group comparison	Raw *p* (Tukey)	Bonferroni adjusted *p* (k=5)	FDR adjusted *q* (BH)	Significant after Bonferroni	Significant after FDR
Non-stress vs CMS + vehicle	<0.001	<0.005	<0.005	Yes	Yes
CMS + vehicle vs CMS + IMP20	<0.001	<0.005	<0.005	Yes	Yes
CMS + vehicle vs CMS + GNR-E 50	<0.001	<0.005	<0.005	Yes	Yes
CMS + vehicle vs CMS + GNR-E 150	<0.001	<0.005	<0.005	Yes	Yes
CMS + vehicle vs CMS + GNR-E 450	<0.001	<0.005	<0.005	Yes	Yes

Table S19. Statistical FDR and Bonferroni analysis of the effect of *Garcinia nigrolineata* resin extract (GNR-E) on CMS-induced hopeless behavior using forced swimming test (FST).

Group comparison	Statistical test	F (df between, df residual)	p-value	Bonferroni-corrected *(p)*	FDR (*q*)
All groups	One-way ANOVA	F(4, 55) = 53.063	< 0.001	< 0.001	< 0.001
Non-stress vs CMS + Vehicle	Tukey's post hoc		< 0.001	< 0.001	< 0.001
CMS + Vehicle vs CMS + IMP (20 mg/kg)	Tukey's post hoc		< 0.001	< 0.001	< 0.001
CMS + Vehicle vs CMS + GNRS (50 mg/kg)	Tukey's post hoc		< 0.001	< 0.001	< 0.001
CMS + Vehicle vs CMS + GNRS (150 mg/kg)	Tukey's post hoc		< 0.001	< 0.001	< 0.001
CMS + Vehicle vs CMS + GNRS (450 mg/kg)	Tukey's post hoc		< 0.001	< 0.001	< 0.001

Multiple-comparison corrections were applied using both Bonferroni and Benjamini–Hochberg FDR methods. All pairwise comparisons remained statistically significant after correction (adjusted $p < 0.001$). Data are expressed as mean ± SEM; n = 12 mice per group.

Table S20. Statistical FDR and Bonferroni analysis of the effect of *Garcinia nigrolineata* resin extract (GNR-E) on CMS-induced hopeless behavior using the tail suspension test (TST).

Group comparison	Statistical test	F (df between, df residual)	p-value	Bonferroni-corrected p	FDR (q)	Adjusted significance / False positive risk
All groups	One-way ANOVA	F(4,55)=53.063	<0.001	<0.001	<0.001	Not false positive
Non-stress vs. CMS + Vehicle	Tukey's post hoc		<0.001	<0.001	<0.001	Not false positive
CMS + Vehicle vs. CMS + IMP (20 mg/kg)	Tukey's post hoc		<0.001	<0.001	<0.001	Not false positive
CMS + Vehicle vs. CMS + GNRS 50 mg/kg	Tukey's post hoc		<0.001	<0.001	<0.001	Not false positive
CMS + Vehicle vs. CMS + GNRS 150 mg/kg	Tukey's post hoc		<0.001	<0.001	<0.001	Not false positive
CMS + Vehicle vs. CMS + GNRS 450 mg/kg	Tukey's post hoc		<0.001	<0.001	<0.001	Not false positive
CMS + GNRS 50 mg/kg vs. CMS + GNRS 450 mg/kg	Tukey's post hoc		0.05	0.35	0.25	Potential false positive (not significant after correction)

Multiple-comparison corrections were performed using both Bonferroni and Benjamini–Hochberg (FDR) procedures. All major comparisons remained significant after correction (adjusted $p < 0.05$), except for CMS + GNRS 50 mg/kg vs. CMS + GNRS 450 mg/kg, which lost significance after adjustment, indicating a possible false positive finding.

Table S21. Statistical FDR and Bonferroni analysis of CMS-induced hypersecretion of the serum corticosterone (CORT) levels.

Group comparison	Statistical test	F (dfbetween, dfresidual)	p-value	Bonferroni-corrected p	FDR (q)	Adjusted significance / False positive risk
All groups	One-way ANOVA	F (4, 15) = 284.816	< 0.001	< 0.001	< 0.001	Not false positive
Non-stress vs. CMS + Vehicle	Tukey's post hoc		< 0.001	< 0.001	< 0.001	Not false positive
CMS + Vehicle vs. CMS + IMP (20 mg/kg)	Tukey's post hoc		< 0.001	< 0.001	< 0.001	Not false positive
CMS + Vehicle vs. CMS + GNRS 50 mg/kg	Tukey's post hoc		< 0.001	< 0.001	< 0.001	Not false positive
CMS + Vehicle vs. CMS + GNRS 150 mg/kg	Tukey's post hoc		< 0.001	< 0.001	< 0.001	Not false positive
CMS + Vehicle vs. CMS + GNRS 450 mg/kg	Tukey's post hoc		< 0.001	< 0.001	< 0.001	Not false positive
CMS + GNRS 50 mg/kg vs. CMS + GNRS 450 mg/kg	Tukey's post hoc		< 0.001	< 0.001	< 0.001	Not false positive

Multiple-comparison corrections were conducted using both Bonferroni and Benjamini–Hochberg (FDR) procedures. All pairwise comparisons remained significant after correction (adjusted $p < 0.05$), indicating no false positive risk among the CORT level data.

Table S22-25. Statistical FDR and Bonferroni analysis of the effect of *Garcinia nigrolineata* resin extract (GNR-E) on CMS-induced changes in serotonin (5-HT) and norepinephrine (NE) levels in frontal cortex and hippocampus.

5-HT level

Frontal cortex

Group comparison	Statistical test	F (dfbetween, dfresidual)	p-value	Bonferroni-corrected p	FDR (q)	Adjusted significance / False positive risk
All groups	One-way ANOVA	F(4,15)=23.154	0.004	0.004	0.004	Not false positive
Non-stress vs. CMS + Vehicle	Tukey's post hoc		<0.001	<0.005	<0.001	Not false positive
CMS + Vehicle vs. CMS + IMP20	Tukey's post hoc		0.018	0.09	0.036	Potential false positive
CMS + Vehicle vs. CMS + GNRS 50	Tukey's post hoc		0.168	0.84	0.168	False positive
CMS + Vehicle vs. CMS + GNRS 150	Tukey's post hoc		0.207	1.035	0.207	False positive
CMS + Vehicle vs. CMS + GNRS 450	Tukey's post hoc		0.002	0.01	0.006	Not false positive

Hippocampus

Group comparison	Statistical test	F (dfbetween, dfresidual)	p-value	Bonferroni-corrected p	FDR (q)	Adjusted significance / False positive risk
All groups	One-way ANOVA	F(4,15)=22.868	<0.001	<0.001	<0.001	Not false positive
Non-stress vs. CMS + Vehicle	Tukey's post hoc		<0.001	<0.005	<0.001	Not false positive
CMS + Vehicle vs. CMS + IMP20	Tukey's post hoc		<0.001	<0.005	<0.001	Not false positive
CMS + Vehicle vs. CMS + GNRS 50	Tukey's post hoc		0.203	1.015	0.203	False positive
CMS + Vehicle vs. CMS + GNRS 150	Tukey's post hoc		0.012	0.06	0.03	Potential false positive
CMS + Vehicle vs. CMS + GNRS 450	Tukey's post hoc		<0.001	<0.005	<0.001	Not false positive
CMS + GNRS 50 vs. CMS + GNRS 450	Tukey's post hoc		<0.001	<0.005	<0.001	Not false positive

NE levels

Frontal cortex

Group comparison	Statistical test	F (dfbetween, dfresidual)	p-value	Bonferroni-corrected p	FDR (q)	Adjusted significance / False positive risk
All groups	One-way ANOVA	F(4,17)=65.963	<0.001	<0.001	<0.001	Not false positive
Non-stress vs. CMS + Vehicle	Tukey's post hoc		<0.001	<0.005	<0.001	Not false positive
CMS + Vehicle vs. CMS + IMP20	Tukey's post hoc		<0.001	<0.005	<0.001	Not false positive
CMS + Vehicle vs. CMS + GNRS 50	Tukey's post hoc		0.069	0.345	0.069	False positive
CMS + Vehicle vs. CMS + GNRS 150	Tukey's post hoc		<0.001	<0.005	<0.001	Not false positive
CMS + Vehicle vs. CMS + GNRS 450	Tukey's post hoc		<0.001	<0.005	<0.001	Not false positive

CMS + GNRS 50 vs. CMS + GNRS 450	Tukey's post hoc		<0.001	<0.005	<0.001	Not false positive
CMS + GNRS 150 vs. CMS + GNRS 450	Tukey's post hoc		0.026	0.13	0.039	Potential false positive

Hippocampus

Group comparison	Statistical test	F (dfbetween, dfresidual)	p-value	Bonferroni-corrected p	FDR (q)	Adjusted significance / False positive risk
All groups	One-way ANOVA	F(4,17)=51.154	<0.001	<0.001	<0.001	Not false positive
Non-stress vs. CMS + Vehicle	Tukey's post hoc		<0.001	<0.005	<0.001	Not false positive
CMS + Vehicle vs. CMS + IMP20	Tukey's post hoc		<0.001	<0.005	<0.001	Not false positive
CMS + Vehicle vs. CMS + GNRS 50	Tukey's post hoc		0.998	4.99	0.998	False positive
CMS + Vehicle vs. CMS + GNRS 150	Tukey's post hoc		0.158	0.79	0.158	False positive
CMS + Vehicle vs. CMS + GNRS 450	Tukey's post hoc		<0.001	<0.005	<0.001	Not false positive
CMS + GNRS 50 vs. CMS + GNRS 450	Tukey's post hoc		<0.001	<0.005	<0.001	Not false positive
CMS + GNRS 150 vs. CMS + GNRS 450	Tukey's post hoc		0.017	0.085	0.034	Potential false positive

Table S26-47. Statistical FDR and Bonferroni analysis of the effect of *Garcinia nigrolineata* resin extract (GNR-E) on CMS-induced changes in serotonin transporter (SERT), serotonin receptor (5HT1A, 5HT1B, 5HT2A, 5HT2C, and 5HT7), norepinephrine transporter (NET), norepinephrine Receptor (α2A, and α2C) and HPA-axis system (GR and SGK-1) biomarkers mRNA expression in frontal cortex and hippocampus.

SERT gene expression

Frontal cortex

Group comparison	Statistical test	F (dfbetween, dfresidual)	p-value	Bonferroni-corrected p	FDR (q)	Adjusted significance / False positive risk
All groups	One-way ANOVA	F(4,25)=14.189	<0.001	<0.001	<0.001	Not false positive
Non-stress vs. CMS + Vehicle	Tukey's post hoc		<0.001	<0.005	<0.001	Not false positive
CMS + Vehicle vs. CMS + IMP20	Tukey's post hoc		<0.001	<0.005	<0.001	Not false positive
CMS + Vehicle vs. CMS + GNRS 50	Tukey's post hoc		0.930	4.65	0.930	False positive
CMS + Vehicle vs. CMS + GNRS 150	Tukey's post hoc		0.404	2.02	0.404	False positive
CMS + Vehicle vs. CMS + GNRS 450	Tukey's post hoc		<0.001	<0.005	<0.001	Not false positive
CMS + GNRS 50 vs. CMS + GNRS 450	Tukey's post hoc		No sig.	-	-	Not significant
CMS + GNRS 150 vs. CMS + GNRS 450	Tukey's post hoc		No sig.	-	-	Not significant

Hippocampus

Group comparison	Statistical test	F (dfbetween, dfresidual)	p-value	Bonferroni-corrected p	FDR (q)	Adjusted significance / False positive risk
All groups	One-way ANOVA	F(4,25)=19.749	<0.001	<0.001	<0.001	Not false positive
Non-stress vs. CMS + Vehicle	Tukey's post hoc		<0.001	<0.005	<0.001	Not false positive
CMS + Vehicle vs. CMS + IMP20	Tukey's post hoc		<0.001	<0.005	<0.001	Not false positive
CMS + Vehicle vs. CMS + GNRS 50	Tukey's post hoc		<0.001	<0.005	<0.005	Not false positive
CMS + Vehicle vs. CMS + GNRS 150	Tukey's post hoc		<0.001	<0.005	<0.005	Not false positive
CMS + Vehicle vs. CMS + GNRS 450	Tukey's post hoc		<0.001	<0.005	<0.005	Not false positive
CMS + GNRS 50 vs. CMS + GNRS 450	Tukey's post hoc		No sig.	-	-	Not significant
CMS + GNRS 150 vs. CMS + GNRS 450	Tukey's post hoc		No sig.	-	-	Not significant

5HT1A gene expression

Frontal cortex

Group comparison	Statistical test	F (dfbetween, dfresidual)	p-value	Bonferroni-corrected p	FDR (q)	Adjusted significance / False positive risk
All groups	One-way ANOVA		<0.001	<0.001	<0.001	Not false positive
Non-stress vs. CMS + Vehicle	Tukey's post hoc		<0.001	<0.005	<0.001	Not false positive
CMS + Vehicle vs. CMS + IMP20	Tukey's post hoc		<0.001	<0.005	<0.001	Not false positive
CMS + Vehicle vs. CMS + GNRS 50	Tukey's post hoc		0.117	0.585	0.117	False positive
CMS + Vehicle vs. CMS + GNRS 150	Tukey's post hoc	F(4,25)=12.755	0.005	0.025	0.010	Not false positive
CMS + Vehicle vs. CMS + GNRS 450	Tukey's post hoc		<0.001	<0.005	<0.001	Not false positive
CMS + GNRS 50 vs. CMS + GNRS 450	Tukey's post hoc		0.033	0.165	0.033	Potential false positive
CMS + GNRS 150 vs. CMS + GNRS 450	Tukey's post hoc		No sig.	-	-	Not significant

Hippocampus

Group comparison	Statistical test	F (dfbetween, dfresidual)	p-value	Bonferroni-corrected p	FDR (q)	Adjusted significance / False positive risk
All groups	One-way ANOVA		0.003	0.015	0.007	Not false positive
Non-stress vs. CMS + Vehicle	Tukey's post hoc		0.002	0.01	0.004	Not false positive
CMS + Vehicle vs. CMS + IMP20	Tukey's post hoc		0.019	0.095	0.038	Potential false positive
CMS + Vehicle vs. CMS + GNRS 50	Tukey's post hoc	F(4,24)=5.259	0.402	2.01	0.402	False positive
CMS + Vehicle vs. CMS + GNRS 150	Tukey's post hoc		0.095	0.475	0.095	False positive
CMS + Vehicle vs. CMS + GNRS 450	Tukey's post hoc		0.003	0.015	0.007	Not false positive

CMS + GNRS 50 vs. CMS + GNRS 450	Tukey's post hoc		No sig.	-	-	Not significant
CMS + GNRS 150 vs. CMS + GNRS 450	Tukey's post hoc		No sig.	-	-	Not significant

5HT1A gene expression

Frontal cortex

Group comparison	Statistical test	F (dfbetween, dfresidual)	p-value	Bonferroni-corrected p	FDR (q)	Adjusted significance / False positive risk
All groups	One-way ANOVA	F(4,25)=16.639	<0.001	<0.001	<0.001	Not false positive
Non-stress vs. CMS + Vehicle	Tukey's post hoc		<0.001	<0.005	<0.001	Not false positive
CMS + Vehicle vs. CMS + IMP20	Tukey's post hoc		<0.001	<0.005	<0.001	Not false positive
CMS + Vehicle vs. CMS + GNRS 50	Tukey's post hoc		<0.001	<0.005	<0.001	Not false positive
CMS + Vehicle vs. CMS + GNRS 150	Tukey's post hoc		0.001	0.005	0.002	Not false positive
CMS + Vehicle vs. CMS + GNRS 450	Tukey's post hoc		<0.001	<0.005	<0.001	Not false positive
CMS + GNRS 50 vs. CMS + GNRS 450	Tukey's post hoc		No sig.	-	-	Not significant
CMS + GNRS 150 vs. CMS + GNRS 450	Tukey's post hoc		No sig.	-	-	Not significant

Hippocampus

Group comparison	Statistical test	F (dfbetween, dfresidual)	p-value	Bonferroni-corrected p	FDR (q)	Adjusted significance / False positive risk
All groups	One-way ANOVA	F(4,25)=8.883	<0.001	<0.001	<0.001	Not false positive
Non-stress vs. CMS + Vehicle	Tukey's post hoc	-	<0.001	<0.005	<0.001	Not false positive
CMS + Vehicle vs. CMS + IMP20	Tukey's post hoc	-	<0.001	<0.005	<0.001	Not false positive
CMS + Vehicle vs. CMS + GNRS 50	Tukey's post hoc	-	0.687	3.435	0.687	False positive
CMS + Vehicle vs. CMS + GNRS 150	Tukey's post hoc	-	0.012	0.06	0.036	Potential false positive
CMS + Vehicle vs. CMS + GNRS 450	Tukey's post hoc	-	0.001	0.005	0.002	Not false positive
CMS + GNRS 50 vs. CMS + GNRS 450	Tukey's post hoc	-	0.032	0.16	0.032	Potential false positive
CMS + GNRS 150 vs. CMS + GNRS 450	Tukey's post hoc	-	0.193	0.965	0.193	False positive

5HT2A gene expression

Frontal cortex

Group comparison	Statistical test	F (dfbetween, dfresidual)	p-value	Bonferroni-corrected p	FDR (q)	Adjusted significance / False positive risk
All groups	One-way ANOVA		0.001	0.005	0.002	Not false positive
Non-stress vs. CMS + Vehicle	Tukey's post hoc		0.003	0.015	0.006	Not false positive

CMS + Vehicle vs. CMS + IMP20	Tukey's post hoc	F(4,25)=6.323	0.007	0.035	0.014	Not false positive
CMS + Vehicle vs. CMS + GNRS 50	Tukey's post hoc		0.977	4.885	0.977	False positive
CMS + Vehicle vs. CMS + GNRS 150	Tukey's post hoc		0.071	0.355	0.071	False positive
CMS + Vehicle vs. CMS + GNRS 450	Tukey's post hoc		0.008	0.04	0.016	Not false positive
CMS + GNRS 50 vs. CMS + GNRS 450	Tukey's post hoc		0.032	0.16	0.032	Potential false positive
CMS + GNRS 150 vs. CMS + GNRS 450	Tukey's post hoc		No sig.	-	-	Not significant

Hippocampus

Group comparison	Statistical test	F (dfbetween, dfresidual)	p-value	Bonferroni-corrected p	FDR (q)	Adjusted significance / False positive risk
All groups	One-way ANOVA	F(4,15)=7.986	<0.001	<0.005	<0.001	Not false positive
Non-stress vs. CMS + Vehicle	Tukey's post hoc		<0.001	<0.005	<0.001	Not false positive
CMS + Vehicle vs. CMS + IMP20	Tukey's post hoc		<0.001	<0.005	<0.001	Not false positive
CMS + Vehicle vs. CMS + GNRS 50	Tukey's post hoc		0.203	1.015	0.203	False positive
CMS + Vehicle vs. CMS + GNRS 150	Tukey's post hoc		<0.001	<0.005	<0.001	Not false positive
CMS + Vehicle vs. CMS + GNRS 450	Tukey's post hoc		<0.001	<0.005	<0.001	Not false positive
CMS + GNRS 50 vs. CMS + GNRS 450	Tukey's post hoc		No sig.	-	-	Not significant
CMS + GNRS 150 vs. CMS + GNRS 450	Tukey's post hoc		No sig.	-	-	Not significant

5HT2C gene expression

Frontal cortex

Group comparison	Statistical test	F (dfbetween, dfresidual)	p-value	Bonferroni-corrected p	FDR (q)	Adjusted significance / False positive risk
All groups	One-way ANOVA	F(4,25)=17.254	<0.001	<0.005	<0.001	Not false positive
Non-stress vs. CMS + Vehicle	Tukey's post hoc		<0.001	<0.005	<0.001	Not false positive
CMS + Vehicle vs. CMS + IMP20	Tukey's post hoc		<0.001	<0.005	<0.001	Not false positive
CMS + Vehicle vs. CMS + GNRS 50	Tukey's post hoc		<0.001	<0.005	<0.001	Not false positive
CMS + Vehicle vs. CMS + GNRS 150	Tukey's post hoc		<0.001	<0.005	<0.001	Not false positive
CMS + Vehicle vs. CMS + GNRS 450	Tukey's post hoc		<0.001	<0.005	<0.001	Not false positive
CMS + GNRS 50 vs. CMS + GNRS 450	Tukey's post hoc		No sig.	-	-	Not significant
CMS + GNRS 150 vs. CMS + GNRS 450	Tukey's post hoc		No sig.	-	-	Not significant

Hippocampus

Group comparison	Statistical test	F (dfbetween, dfresidual)	p-value	Bonferroni-corrected p	FDR (q)	Adjusted significance / False positive risk
All groups	One-way ANOVA	F(4,25)=15.549	<0.001	<0.005	<0.001	Not false positive
Non-stress vs. CMS + Vehicle	Tukey's post hoc		<0.001	<0.005	<0.001	Not false positive
CMS + Vehicle vs. CMS + IMP20	Tukey's post hoc		<0.001	<0.005	<0.001	Not false positive
CMS + Vehicle vs. CMS + GNRS 50	Tukey's post hoc		0.093	0.465	0.093	False positive
CMS + Vehicle vs. CMS + GNRS 150	Tukey's post hoc		0.006	0.03	0.012	Not false positive
CMS + Vehicle vs. CMS + GNRS 450	Tukey's post hoc		<0.001	<0.005	<0.001	Not false positive
CMS + GNRS 50 vs. CMS + GNRS 450	Tukey's post hoc		0.006	0.03	0.012	Not false positive
CMS + GNRS 150 vs. CMS + GNRS 450	Tukey's post hoc		No sig.	-	-	Not significant

5HT7 gene expression

Frontal cortex

Group comparison	Statistical test	F (dfbetween, dfresidual)	p-value	Bonferroni-corrected p	FDR (q)	Adjusted significance / False positive risk
All groups	One-way ANOVA	F(4,25)=9.964	<0.001	<0.005	<0.001	Not false positive
Non-stress vs. CMS + Vehicle	Tukey's post hoc		<0.001	<0.005	<0.001	Not false positive
CMS + Vehicle vs. CMS + IMP20	Tukey's post hoc		<0.001	<0.005	<0.001	Not false positive
CMS + Vehicle vs. CMS + GNRS 50	Tukey's post hoc		0.003	0.015	0.006	Not false positive
CMS + Vehicle vs. CMS + GNRS 150	Tukey's post hoc		0.037	0.185	0.037	Potential false positive
CMS + Vehicle vs. CMS + GNRS 450	Tukey's post hoc		<0.001	<0.005	<0.001	Not false positive
CMS + GNRS 50 vs. CMS + GNRS 450	Tukey's post hoc		No sig.	-	-	Not significant
CMS + GNRS 150 vs. CMS + GNRS 450	Tukey's post hoc		No sig.	-	-	Not significant

Hippocampus

Group comparison	Statistical test	F (dfbetween, dfresidual)	p-value	Bonferroni-corrected p	FDR (q)	Adjusted significance / False positive risk
All groups	One-way ANOVA	F(4,25)=8.285	<0.001	<0.005	<0.001	Not false positive
Non-stress vs. CMS + Vehicle	Tukey's post hoc		<0.001	<0.005	<0.001	Not false positive
CMS + Vehicle vs. CMS + IMP20	Tukey's post hoc		<0.001	<0.005	<0.001	Not false positive
CMS + Vehicle vs. CMS + GNRS 50	Tukey's post hoc		0.178	0.89	0.178	False positive
CMS + Vehicle vs. CMS + GNRS 150	Tukey's post hoc		0.003	0.015	0.006	Not false positive
CMS + Vehicle vs. CMS + GNRS 450	Tukey's post hoc		<0.001	<0.005	<0.001	Not false positive

CMS + GNRS 50 vs. CMS + GNRS 450	Tukey's post hoc		0.001	0.005	0.002	Not false positive
CMS + GNRS 150 vs. CMS + GNRS 450	Tukey's post hoc		No sig.	-	-	Not significant

NET gene expression

Frontal cortex

Group comparison	Statistical test	F (dfbetween, dfresidual)	p-value	Bonferroni-corrected p	FDR (q)	Adjusted significance / False positive risk
All groups	One-way ANOVA	F(4,25)=24.220	<0.001	<0.005	<0.001	Not false positive
Non-stress vs. CMS + Vehicle	Tukey's post hoc		<0.001	<0.005	<0.001	Not false positive
CMS + Vehicle vs. CMS + IMP20	Tukey's post hoc		<0.001	<0.005	<0.001	Not false positive
CMS + Vehicle vs. CMS + GNRS 50	Tukey's post hoc		0.984	4.92	0.984	False positive
CMS + Vehicle vs. CMS + GNRS 150	Tukey's post hoc		0.093	0.465	0.093	False positive
CMS + Vehicle vs. CMS + GNRS 450	Tukey's post hoc		<0.001	<0.005	<0.001	Not false positive
CMS + GNRS 50 vs. CMS + GNRS 450	Tukey's post hoc		<0.001	<0.005	<0.001	Not false positive
CMS + GNRS 150 vs. CMS + GNRS 450	Tukey's post hoc		No sig.	-	-	Not significant

Hippocampus

Group comparison	Statistical test	F (dfbetween, dfresidual)	p-value	Bonferroni-corrected p	FDR (q)	Adjusted significance / False positive risk
All groups	One-way ANOVA	F(4,25)=18.640	<0.001	<0.005	<0.001	Not false positive
Non-stress vs. CMS + Vehicle	Tukey's post hoc		<0.001	<0.005	<0.001	Not false positive
CMS + Vehicle vs. CMS + IMP20	Tukey's post hoc		<0.001	<0.005	<0.001	Not false positive
CMS + Vehicle vs. CMS + GNRS 50	Tukey's post hoc		0.966	4.83	0.966	False positive
CMS + Vehicle vs. CMS + GNRS 150	Tukey's post hoc		0.039	0.195	0.039	Potential false positive
CMS + Vehicle vs. CMS + GNRS 450	Tukey's post hoc		<0.001	<0.005	<0.001	Not false positive
CMS + GNRS 50 vs. CMS + GNRS 450	Tukey's post hoc		0.001	0.005	0.002	Not false positive
CMS + GNRS 150 vs. CMS + GNRS 450	Tukey's post hoc		No sig.	-	-	Not significant

α2A gene expression

Frontal cortex

Group comparison	Statistical test	F (dfbetween, dfresidual)	p-value	Bonferroni-corrected p	FDR (q)	Adjusted significance / False positive risk
All groups	One-way ANOVA	F(4,25)=27.652	<0.001	<0.005	<0.001	Not false positive
Non-stress vs. CMS + Vehicle	Tukey's post hoc		<0.001	<0.005	<0.001	Not false positive

CMS + Vehicle vs. CMS + IMP20	Tukey's post hoc		<0.001	<0.005	<0.001	Not false positive
CMS + Vehicle vs. CMS + GNRS 50	Tukey's post hoc		0.298	1.49	0.298	False positive
CMS + Vehicle vs. CMS + GNRS 150	Tukey's post hoc		<0.001	<0.005	<0.001	Not false positive
CMS + Vehicle vs. CMS + GNRS 450	Tukey's post hoc		<0.001	<0.005	<0.001	Not false positive
CMS + GNRS 50 vs. CMS + GNRS 450	Tukey's post hoc		No sig.	-	-	Not significant
CMS + GNRS 50 vs. CMS + GNRS 450	Tukey's post hoc		<0.001	<0.005	<0.001	Not false positive

Hippocampus

Group comparison	Statistical test	F (dfbetween, dfresidual)	p-value	Bonferroni-corrected p	FDR (q)	Adjusted significance / False positive risk
All groups	One-way ANOVA	F(4,25)=87.633	<0.001	<0.005	<0.001	Not false positive
Non-stress vs. CMS + Vehicle	Tukey's post hoc		<0.001	<0.005	<0.001	Not false positive
CMS + Vehicle vs. CMS + IMP20	Tukey's post hoc		<0.001	<0.005	<0.001	Not false positive
CMS + Vehicle vs. CMS + GNRS 50	Tukey's post hoc		0.002	0.01	0.004	Not false positive
CMS + Vehicle vs. CMS + GNRS 150	Tukey's post hoc		<0.001	<0.005	<0.001	Not false positive
CMS + Vehicle vs. CMS + GNRS 450	Tukey's post hoc		<0.001	<0.005	<0.001	Not false positive
CMS + GNRS 50 vs. CMS + GNRS 450	Tukey's post hoc		<0.001	<0.005	0.001	Not false positive
CMS + GNRS 150 vs. CMS + GNRS 450	Tukey's post hoc		<0.001	<0.005	0.001	Not false positive
CMS + GNRS 50 vs. CMS + GNRS 150	Tukey's post hoc		<0.001	<0.005	0.001	Not false positive

α2C gene expression

Frontal cortex

Group comparison	Statistical test	F (dfbetween, dfresidual)	p-value	Bonferroni-corrected p	FDR (q)	Adjusted significance / False positive risk
All groups	One-way ANOVA	F(4,25)=50.751	<0.001	<0.005	<0.001	Not false positive
Non-stress vs. CMS + Vehicle	Tukey's post hoc		<0.001	<0.005	<0.001	Not false positive
CMS + Vehicle vs. CMS + IMP20	Tukey's post hoc		<0.001	<0.005	<0.001	Not false positive
CMS + Vehicle vs. CMS + GNRS 50	Tukey's post hoc		<0.001	<0.005	<0.001	Not false positive
CMS + Vehicle vs. CMS + GNRS 150	Tukey's post hoc		<0.001	<0.005	<0.001	Not false positive
CMS + Vehicle vs. CMS + GNRS 450	Tukey's post hoc		<0.001	<0.005	<0.001	Not false positive
CMS + GNRS 50 vs. CMS + GNRS 450	Tukey's post hoc		<0.001	<0.005	<0.001	Not false positive
CMS + GNRS 150 vs. CMS + GNRS 450	Tukey's post hoc		No sig.	-	-	Not significant

Hippocampus

Group comparison	Statistical test	F (dfbetween, dfresidual)	p-value	Bonferroni-corrected p	FDR (q)	Adjusted significance / False positive risk
All groups	One-way ANOVA		<0.001	<0.005	<0.001	Not false positive
Non-stress vs. CMS + Vehicle	Tukey's post hoc		<0.001	<0.005	<0.001	Not false positive
CMS + Vehicle vs. CMS + IMP20	Tukey's post hoc		<0.001	<0.005	<0.001	Not false positive
CMS + Vehicle vs. CMS + GNRS 50	Tukey's post hoc	F(4,12)=9.850	0.951	4.755	0.951	False positive
CMS + Vehicle vs. CMS + GNRS 150	Tukey's post hoc		0.384	1.92	0.384	False positive
CMS + Vehicle vs. CMS + GNRS 450	Tukey's post hoc		0.013	0.065	0.026	Not false positive
CMS + GNRS 50 vs. CMS + GNRS 450	Tukey's post hoc		No sig.	-	-	Not significant
CMS + GNRS 150 vs. CMS + GNRS 450	Tukey's post hoc		No sig.	-	-	Not significant

GR gene expression

Frontal cortex

Group comparison	Statistical test	F (dfbetween, dfresidual)	p-value	Bonferroni-corrected p	FDR (q)	Adjusted significance / False positive risk
All groups	One-way ANOVA		<0.001	<0.005	<0.001	Not false positive
Non-stress vs. CMS + Vehicle	Tukey's post hoc		<0.001	<0.005	<0.001	Not false positive
CMS + Vehicle vs. CMS + IMP20	Tukey's post hoc		<0.001	<0.005	<0.001	Not false positive
CMS + Vehicle vs. CMS + GNRS 50	Tukey's post hoc	F(4,25)=55.901	<0.001	<0.005	<0.001	Not false positive
CMS + Vehicle vs. CMS + GNRS 150	Tukey's post hoc		<0.001	<0.005	<0.001	Not false positive
CMS + Vehicle vs. CMS + GNRS 450	Tukey's post hoc		<0.001	<0.005	<0.001	Not false positive
CMS + GNRS 50 vs. CMS + GNRS 450	Tukey's post hoc		<0.001	<0.005	<0.001	Not false positive
CMS + GNRS 150 vs. CMS + GNRS 450	Tukey's post hoc		No sig.	-	-	Not significant

Hippocampus

Group comparison	Statistical test	F (dfbetween, dfresidual)	p-value	Bonferroni-corrected p	FDR (q)	Adjusted significance / False positive risk
All groups	One-way ANOVA		<0.001	<0.005	<0.001	Not false positive
Non-stress vs. CMS + Vehicle	Tukey's post hoc		<0.001	<0.005	<0.001	Not false positive
CMS + Vehicle vs. CMS + IMP20	Tukey's post hoc		<0.001	<0.005	<0.001	Not false positive
CMS + Vehicle vs. CMS + GNRS 50	Tukey's post hoc	F(4,25)=30.098	0.012	0.06	0.024	Not false positive
CMS + Vehicle vs. CMS + GNRS 150	Tukey's post hoc		<0.001	<0.005	<0.001	Not false positive
CMS + Vehicle vs. CMS + GNRS 450	Tukey's post hoc		<0.001	<0.005	<0.001	Not false positive
CMS + GNRS 50 vs. CMS + GNRS 450	Tukey's post hoc		<0.001	<0.005	0.001	Not false positive

CMS + GNRS 150 vs. CMS + GNRS 450	Tukey's post hoc		No sig.	-	-	Not significant

SGK-1 gene expression

Frontal cortex

Group comparison	Statistical test	F (dfbetween, dfresidual)	p-value	Bonferroni-corrected p	FDR (q)	Adjusted significance / False positive risk
All groups	One-way ANOVA	F(4,25)=21.138	<0.001	<0.005	<0.001	Not false positive
Non-stress vs. CMS + Vehicle	Tukey's post hoc		<0.001	<0.005	<0.001	Not false positive
CMS + Vehicle vs. CMS + IMP20	Tukey's post hoc		<0.001	<0.005	<0.001	Not false positive
CMS + Vehicle vs. CMS + GNRS 50	Tukey's post hoc		0.742	3.71	0.742	False positive
CMS + Vehicle vs. CMS + GNRS 150	Tukey's post hoc		0.003	0.015	0.006	Not false positive
CMS + Vehicle vs. CMS + GNRS 450	Tukey's post hoc		<0.001	<0.005	<0.001	Not false positive
CMS + GNRS 50 vs. CMS + GNRS 450	Tukey's post hoc		<0.001	<0.005	0.001	Not false positive
CMS + GNRS 150 vs. CMS + GNRS 450	Tukey's post hoc		No sig.	-	-	Not significant

Hippocampus

Group comparison	Statistical test	F (dfbetween, dfresidual)	p-value	Bonferroni-corrected p	FDR (q)	Adjusted significance / False positive risk
All groups	One-way ANOVA	F(4,25)=25.837	<0.001	<0.005	<0.001	Not false positive
Non-stress vs. CMS + Vehicle	Tukey's post hoc		<0.001	<0.005	<0.001	Not false positive
CMS + Vehicle vs. CMS + IMP20	Tukey's post hoc		<0.001	<0.005	<0.001	Not false positive
CMS + Vehicle vs. CMS + GNRS 50	Tukey's post hoc		0.131	0.655	0.131	False positive
CMS + Vehicle vs. CMS + GNRS 150	Tukey's post hoc		<0.001	<0.005	<0.001	Not false positive
CMS + Vehicle vs. CMS + GNRS 450	Tukey's post hoc		<0.001	<0.005	<0.001	Not false positive
CMS + GNRS 50 vs. CMS + GNRS 450	Tukey's post hoc		<0.001	<0.005	0.001	Not false positive
CMS + GNRS 150 vs. CMS + GNRS 450	Tukey's post hoc		No sig.	-	-	Not significant
CMS + GNRS 50 vs. CMS + GNRS 150	Tukey's post hoc		0.013	0.065	0.026	Not false positive

Power of analysis

Table S48. Effect size (partial η^2), Cohen's f, and achieved statistical power ($1-\beta$) for monoaminergic, receptor, and glucocorticoid-related markers in the frontal cortex and hippocampus.

Achieved power calculated from partial η^2, converted to Cohen's $f = \sqrt{\eta^2/(1-\eta^2)}$ for one-way ANOVA ($\alpha = 0.05$). Power ≥ 0.8 = adequate.

Variable	Brain region	dfbetween	dfresidual	Partial η^2	Cohen's f	Achieved power (1−β)	Interpretation
5-HT	Frontal cortex	4	15	0.86	2.48	1.00	Adequate
5-HT	Hippocampus	4	15	0.86	2.48	1.00	Adequate
NE	Frontal cortex	4	17	0.94	3.77	1.00	Adequate
NE	Hippocampus	4	17	0.92	3.36	1.00	Adequate
SERT	Frontal cortex	4	25	0.69	1.48	0.99	Adequate
SERT	Hippocampus	4	25	0.76	1.78	0.99	Adequate
5-HT1A	Frontal cortex	4	25	0.67	1.45	0.98	Adequate
5-HT1A	Hippocampus	4	24	0.47	0.94	0.87	Adequate
5-HT1B	Frontal cortex	4	25	0.73	1.63	0.99	Adequate
5-HT1B	Hippocampus	4	25	0.59	1.20	0.95	Adequate
5-HT2A	Frontal cortex	4	25	0.50	1.00	0.92	Adequate
5-HT2A	Hippocampus	4	15	0.68	1.49	0.98	Adequate
5-HT2C	Frontal cortex	4	25	0.73	1.63	0.99	Adequate
5-HT2C	Hippocampus	4	25	0.71	1.57	0.99	Adequate
5-HT7	Frontal cortex	4	25	0.61	1.25	0.97	Adequate
5-HT7	Hippocampus	4	25	0.57	1.14	0.96	Adequate
NET	Frontal cortex	4	25	0.80	2.00	1.00	Adequate
NET	Hippocampus	4	25	0.75	1.73	0.99	Adequate
α2A	Frontal cortex	4	25	0.82	2.13	1.00	Adequate
α2A	Hippocampus	4	25	0.93	3.69	1.00	Adequate
α2C	Frontal cortex	4	25	0.89	2.84	1.00	Adequate
α2C	Hippocampus	4	12	0.77	1.85	0.98	Adequate
GR	Frontal cortex	4	25	0.90	3.00	1.00	Adequate
GR	Hippocampus	4	25	0.83	2.09	0.99	Adequate
SGK-1	Frontal cortex	4	25	0.77	1.85	0.98	Adequate
SGK-1	Hippocampus	4	25	0.81	1.96	0.99	Adequate

Note:

- Partial η^2 (eta squared) represents the proportion of variance explained by the treatment effect, controlling for other factors.
- Cohen's f is derived from partial η^2 using the formula $f = \sqrt{\eta^2 / (1 - \eta^2)}$, and represents the standardized effect size.
- The achieved power (1−β) indicates the probability of correctly rejecting the null hypothesis.
- Interpretation thresholds follow Cohen's conventional criteria:
  - Partial η^2: small = 0.01, medium = 0.06, large ≥ 0.14
  - Cohen's f: small = 0.10, medium = 0.25, large ≥ 0.40
  - Power ≥ 0.80 is considered adequate.

All tested variables demonstrated large effect sizes (partial $\eta^2 > 0.14$) and adequate power (≥ 0.80), supporting the robustness of the statistical results.

Effect size analysis

Table S49. Effect size (partial η²) of biological relevance in Frontal Cortex and Hippocampus.

Effect size (partial η^2) calculated from ANOVA results. Values of F, df, and p are reported for each group comparison. Effect size interpretation: small ($\eta^2 = 0.01$), medium ($\eta^2 = 0.06$), large ($\eta^2 = 0.14$). Sample size: N=6 per group; note that this may limit statistical power for detecting smaller effects.

Biological relevance	Brain Region	F (df_between, df_residual)	p-value	Partial η^2	Effect Size
5HT	Frontal Cortex	23.154 (4,15)	0.004	0.86	Large
5HT	Hippocampus	22.868 (4,15)	<0.001	0.86	Large
NE	Frontal Cortex	65.963 (4,17)	<0.001	0.94	Large
NE	Hippocampus	51.154 (4,17)	<0.001	0.92	Large
SERT	Frontal Cortex	14.189 (4,25)	<0.001	0.69	Large
SERT	Hippocampus	19.749 (4,25)	<0.001	0.76	Large
5HT1A	Frontal Cortex	12.755 (4,25)	<0.001	0.67	Large
5HT1A	Hippocampus	5.259 (4,24)	0.003	0.47	Large
5HT1B	Frontal Cortex	16.639 (4,25)	<0.001	0.73	Large
5HT1B	Hippocampus	8.883 (4,25)	<0.001	0.59	Large
5HT2A	Frontal Cortex	6.323 (4,25)	0.001	0.50	Large
5HT2A	Hippocampus	7.986 (4,15)	<0.001	0.68	Large
5HT2C	Frontal Cortex	17.254 (4,25)	<0.001	0.73	Large
5HT2C	Hippocampus	15.549 (4,25)	<0.001	0.71	Large
5HT7	Frontal Cortex	9.964 (4,25)	<0.001	0.61	Large
5HT7	Hippocampus	8.285 (4,25)	<0.001	0.57	Large
NET	Frontal Cortex	24.220 (4,25)	<0.001	0.80	Large
NET	Hippocampus	18.640 (4,25)	<0.001	0.75	Large
α2A	Frontal Cortex	27.652 (4,25)	<0.001	0.82	Large
α2A	Hippocampus	87.633 (4,25)	<0.001	0.93	Large
α2C	Frontal Cortex	50.751 (4,25)	<0.001	0.89	Large
α2C	Hippocampus	9.850 (4,12)	<0.001	0.77	Large
GR	Frontal Cortex	55.901 (4,25)	<0.001	0.90	Large
GR	Hippocampus	30.098 (4,25)	<0.001	0.83	Large
SGK-1	Frontal Cortex	21.138 (4,25)	<0.001	0.77	Large
SGK-1	Hippocampus	25.837 (4,25)	<0.001	0.81	Large

Note: Partial η^2 was calculated using the formula $\eta^2 = (F \times df\_between) / (F \times df\_between + df\_residual)$. Effect size interpretation: small ($\eta^2 = 0.01$), medium ($\eta^2 = 0.06$), large ($\eta^2 = 0.14$). Sample size per group is N=6; caution is advised as small N may limit power to detect smaller effects.
